# RNA-binding properties orchestrate TDP-43 homeostasis through condensate formation *in vivo*

**DOI:** 10.1093/nar/gkae112

**Published:** 2024-02-21

**Authors:** Natalie M Scherer, Cindy Maurel, Matthew S Graus, Luke McAlary, Grant Richter, Rowan A W Radford, Alison Hogan, Emily K Don, Albert Lee, Justin Yerbury, Mathias Francois, Roger S Chung, Marco Morsch

**Affiliations:** Faculty of Medicine, Health & Human Sciences, Macquarie Medical School, MND Research Centre, Macquarie University, Sydney, NSW 2109, Australia; Faculty of Medicine, Health & Human Sciences, Macquarie Medical School, MND Research Centre, Macquarie University, Sydney, NSW 2109, Australia; The David Richmond Laboratory for Cardio-Vascular Development: gene regulation and editing, Centenary Institute, The University of Sydney, School of Medical Sciences, Sydney, NSW 2006, Australia; Genome Imaging Centre, Centenary Institute, The University of Sydney, Sydney, NSW 2006, Australia; Molecular Horizons and School of Chemistry and Molecular Bioscience, University of Wollongong, Wollongong, NSW 2522, Australia; Faculty of Medicine, Health & Human Sciences, Macquarie Medical School, MND Research Centre, Macquarie University, Sydney, NSW 2109, Australia; Faculty of Medicine, Health & Human Sciences, Macquarie Medical School, MND Research Centre, Macquarie University, Sydney, NSW 2109, Australia; Faculty of Medicine, Health & Human Sciences, Macquarie Medical School, MND Research Centre, Macquarie University, Sydney, NSW 2109, Australia; Faculty of Medicine, Health & Human Sciences, Macquarie Medical School, MND Research Centre, Macquarie University, Sydney, NSW 2109, Australia; Faculty of Medicine, Health & Human Sciences, Macquarie Medical School, MND Research Centre, Macquarie University, Sydney, NSW 2109, Australia; Molecular Horizons and School of Chemistry and Molecular Bioscience, University of Wollongong, Wollongong, NSW 2522, Australia; The David Richmond Laboratory for Cardio-Vascular Development: gene regulation and editing, Centenary Institute, The University of Sydney, School of Medical Sciences, Sydney, NSW 2006, Australia; Genome Imaging Centre, Centenary Institute, The University of Sydney, Sydney, NSW 2006, Australia; Faculty of Medicine, Health & Human Sciences, Macquarie Medical School, MND Research Centre, Macquarie University, Sydney, NSW 2109, Australia; Faculty of Medicine, Health & Human Sciences, Macquarie Medical School, MND Research Centre, Macquarie University, Sydney, NSW 2109, Australia

## Abstract

Insoluble cytoplasmic aggregate formation of the RNA-binding protein TDP-43 is a major hallmark of neurodegenerative diseases including Amyotrophic Lateral Sclerosis. TDP-43 localizes predominantly in the nucleus, arranging itself into dynamic condensates through liquid–liquid phase separation (LLPS). Mutations and post-translational modifications can alter the condensation properties of TDP-43, contributing to the transition of liquid-like biomolecular condensates into solid-like aggregates. However, to date it has been a challenge to study the dynamics of this process *in vivo*. We demonstrate through live imaging that human TDP-43 undergoes nuclear condensation in spinal motor neurons in a living animal. RNA-binding deficiencies as well as post-translational modifications can lead to aberrant condensation and altered TDP-43 compartmentalization. Single-molecule tracking revealed an altered mobility profile for RNA-binding deficient TDP-43. Overall, these results provide a critically needed *in vivo* characterization of TDP-43 condensation, demonstrate phase separation as an important regulatory mechanism of TDP-43 accessibility, and identify a molecular mechanism of how functional TDP-43 can be regulated.

## Graphical Abstract

**Graphical Abstract:**
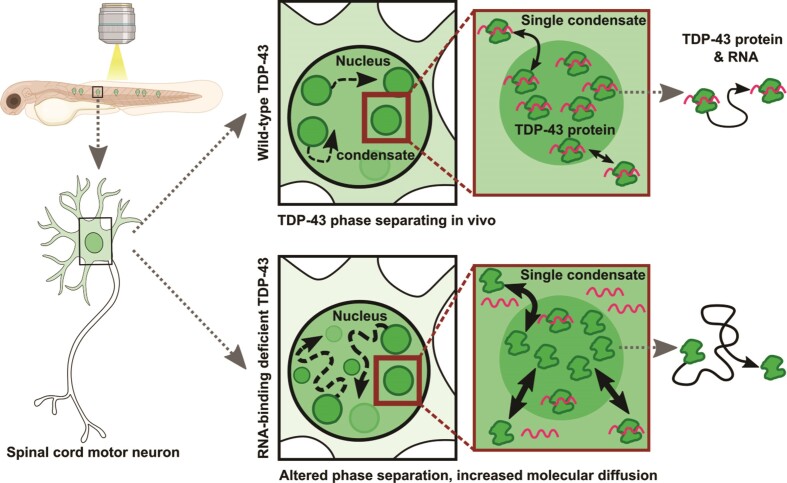

## Introduction

TAR DNA-binding protein 43 (TDP-43) is a DNA- and RNA-binding protein that predominantly localizes in the nucleus but can shuttle back and forth into the cytoplasm. The cytoplasmic mislocalization and formation of insoluble aggregates of TDP-43 are hallmark pathological features in neurodegenerative diseases including amyotrophic lateral sclerosis (ALS), limbic predominant age-related TDP-43 encephalopathy (LATE), frontotemporal lobar dementia (FTLD), and to a lesser degree in Alzheimer's disease (AD) (1–4). While identifying the underlying molecular processes of cytoplasmic mislocalization and aggregation of TDP-43 is highly relevant for understanding neurodegenerative disease pathogenesis, it is also important to better understand the regulatory mechanisms that maintain functional TDP-43 levels within the nucleus.

Physiologically, TDP-43 has the capacity to self-accumulate and concentrate into droplet-like structures in the nucleus *in vitro* (5–9) and spinal motor neurons *in vivo* (10). The process that underpins this accumulation into biomolecular condensates (BMCs) is termed liquid-liquid phase separation (LLPS). BMCs are liquid-like membrane-less organelles with the capacity to fuse and separate spontaneously (11). LLPS recently gained significant interest as it contributes to the spatiotemporal maintenance of protein compartmentalization, organization and function (12). Non-specific, low affinity, and specific interactions act in concert to govern and regulate LLPS (13) and are dependent on the physicochemical properties of the protein sequence (14). TDP-43 can move in and out of BMCs to fulfill its role in biological processes such as RNA translation and gene expression regulation (15,16). Structural motifs that are important for TDP-43 LLPS include (i) the N-terminal domain (NTD_1-76_) enabling self-oligomerization (17,18), (ii) two RNA Recognition Motifs (RRM1_106–176_ and RRM2_191–259_) that can stabilize the protein through RNA-binding (7,19) and (iii) a C-terminal disordered or low-complexity domain (LCD), often referred to as prion-like domain due to its sequence similarity to the yeast prion-protein (20,21). ALS-linked mutations in the LCD of TDP-43 have been reported to result in structural changes including aberrant phase transition from a liquid- to a solid-like state, likely contributing to the formation of pathological aggregates in the cytoplasm (20,22,23). However, evidence of whether condensate formation of TDP-43 in the nucleus is contributing to the physiological regulation and homeostasis of TDP-43 itself is just beginning to emerge (18,24) and dynamic characterization of nuclear condensates has been lacking *in vivo*.

Through its two RRMs, critical for DNA and RNA interactions (25–27), TDP-43 binds hundreds of different RNAs with high affinity to UG-repeats, predominantly found in long introns, the 3′untranslated region (3′UTR) of genes, or in non-coding RNAs (26,28). TDP-43, therefore, plays a crucial role in the regulation of RNA splicing, stability, transport, and translation, and thus contributes to maintaining essential cellular functions (29–34). The RRMs in TDP-43 also harbor multiple sites important for post-translational modifications, such as lysine acetylation (e.g. K121, K136, K145, K192) (5,35–37). Acetylation of such lysine residues removes their positive charge and therefore, their ability to undergo charge-mediated interactions with negatively charged nucleic acids (38), consequently reducing TDP-43′s capacity to bind RNA (5). *In vitro* studies revealed that the acetylation-mimicking TDP-43 variant TDP-43^K145Q, K192Q^ (TDP-43 2KQ with lysine to glutamine substitutions) showed 50–65% reduced RNA-binding capacity. Another TDP-43 variant with phenylalanine to leucine substitutions, TDP-43^F147L, F149L, F229L, F231L^ (TDP-43 4FL), showed 70% RNA-binding deficiency (5). These TDP-43 variants also revealed enhanced nuclear phase separation (5,9,39) and, when expressed in the cytoplasm, TDP-43 was found to be aggregated, hyperphosphorylated, and ubiquitinated (5,8,9). Ubiquitination and phosphorylation have also been identified to play a role in altered BMC characteristics and liquid-to-solid phase transition in the cytoplasm (5,8).

Despite a body of *in vitro* studies on how mutations, PTMs or RNA-binding can affect TDP-43 localization and compartmentalization, there remains limited *in vivo* understanding of the molecular drivers of TDP-43 dynamism across the spectrum of functional oligomers, phase separated condensates and pathological aggregates (12,40,41)—raising questions about the physiological relevance in more complex biological systems. In this study, we established a zebrafish platform to characterize the dynamic condensation behavior of nuclear TDP-43 and its cellular consequences *in vivo*. We demonstrate that nuclear TDP-43 fulfils critical LLPS criteria when expressed *in vivo* in zebrafish spinal cord motor neurons, such as the formation of spherical, liquid-like biomolecular condensates, which can spontaneously undergo fusion and fission. To our surprise, the ALS-linked mutations G294V and Q331K in the low-complexity domain of TDP-43 did not result in changes of nuclear condensation characteristics compared to wildtype TDP-43. However, a reduction in the RNA-binding ability of TDP-43 resulted in decreased cytoplasmic expression levels, enhanced nuclear phase separation, and faster fluorescence recovery *in vivo* and *in vitro*. Single-molecule tracking of TDP-43 revealed that an increased mobility profile of RNA-binding deficient TDP-43 might be the underlying driver of these changes. Overall, the results highlight the importance of RNA-binding in regulating nuclear condensation of TDP-43 *in vivo* and demonstrate a molecular mechanism of how condensation can regulate TDP-43 accessibility and may drive pathologically relevant changes.

## Materials and methods

### Ethics statements

All *in vivo* experiments using zebrafish (*Danio rerio*) were approved by Macquarie University Animal Ethics and Biosafety committees (ARA (2017/19, 2015/033), biosafety approval (NLRD 5974: 52019597412350). The *in vitro* experiments were approved via the UOW Institutional Biosafety Committee (UOW IBC2203).

### Zebrafish lines and husbandry

All zebrafish were maintained under standard conditions in a facility at Macquarie University. Embryos were collected from natural mating of adult zebrafish and raised at 28°C in E3 solution (5 mM NaCl, 0.17 mM KCl, 0.33 mM CaCl_2_ and 0.33 mM MgSO_4_ buffered to 7.3 pH using carbonate hardness generator (Aquasonic), no methylene blue). All experimental procedures were performed in the timespan of 3–5 days post fertilization (dpf) and under anesthesia using 0.01% (w/v) tricaine methansulfonate (MS-222, Sigma).

Zebrafish lines used for *in vivo* experiments were on AB/Tuebingen (TAB) wildtype background or transgenic lines Tg(-3mnx1:Hsa.H2B-mCerulean3), Tg(-3mnx1:mTagBFP), Tg(-3mnx1:eGFP-Hsa.TDP-43^WT^), Tg(-3mnx1:eGFP-Hsa.TDP-43^G294V^), Tg(-3mnx1:eGFP-Hsa.TDP-43^dNLS^) and Tg(-3mnx1:eGFP-Hsa.TDP-43^2KQ^).

### Cell lines and culture conditions

U2OS cells were maintained under 5% atmospheric CO_2_ in humidified incubation chambers at 37°C in DMEM/F12 medium supplemented with 10% fetal bovine serum and 2 mM l-glutamine. HEK-293 cells and HeLa cells were cultured in DMEM (Gibco) supplemented with 10% FBS, 1% GlutaMAX (Gibco) and 1% MEM non-essential amino acids (MEM NEAA, Gibco). Cells were maintained at 37°C with 5% CO_2_.

### *In vivo* experiments

#### Generation of mosaic zebrafish embryos and stable transgenic fish lines

To create the transient zebrafish lines used in this study, the following DNA constructs were microinjected (1uL of a mix containing 25 ng/μl plasmid DNA) into the zebrafish zygote at the one-cell stage of development: -3mnx1:eGFP-Hsa.TDP-43^WT^ (referred to as hTDP-43^WT^), -3mnx1:eGFP-Hsa.TDP-43^G294V^ (referred to as hTDP-43^G294V^), -3mnx1:eGFP-Hsa.TDP-43^dNLS^ (referred to as hTDP-43 ^dNLS^), -3mnx1:eGFP-Hsa.TDP-43^K145Q, K192Q^ (referred to as hTDP-43 ^2KQ^) and -3mnx1:eGFP-Hsa.TDP-43^F147L, F149L, F229L, F231L^ (referred to as hTDP-43 ^4FL^). To create the stable transgenic fish lines *Tg(-3mnx1:eGFP-Hsa.TDP-43 ^WT^; mq19Tg), Tg(-3mnx1:eGFP-Hsa.TDP-43 ^G294V^; mq20Tg), Tg(-3mnx1:eGFP-Hsa.TDP-43 ^dNLS^*; mq21Tg) and *Tg(-3mnx1:eGFP-Hsa.TDP-43^2KQ^; mq22Tg)* which were used for western blotting and the stable transgenic fish line *Tg*(*-3mnx1:Hsa.H2B-mCerulean3;*mq18Tg) used for some *in vivo* experiments, the respective DNA constructs and Tol2 transposase mRNA (25 ng/μl) (42) were co-injected into the zebrafish zygote at the one-cell stage of development. Embryos expressing the desired DNA construct were selected at 3 dpf and raised to sexual maturity and screened for germ line incorporation.

#### Generation of DNA constructs

The final DNA constructs were generated using the Multisite Gateway^®^ Three-Fragment Vector Construction Kit (43) into recombined pTol2pA2 vector (43) as destination vector with p5E-3mnx1 (44), pME-eGFPns (45) and p3E-Hsa.TDP-43 (WT or mutant version) (10). Subsequent site directed mutagenesis was performed by GenScript and the following mutations were introduced to the cDNA sequence of the p3E-Hsa.TDP-43 WT construct. For p3E-Hsa.TDP-43 G294V, GGG > GTT; for p3E-Hsa.TDP-43 delta NLS (K82A, R83A, K84A), AAAAGAAAA > GCTGCTGCT; for p3E Hsa.TDP-43 2KQ (K145Q, K192Q), AAG > CAA and AAA > CAA; for p3E Hsa.TDP-43 4FL (F147L, F149L F,229L, F231L), TTT > CTT; for p3E Hsa.TDP-43 Q331K, CAG > AAG. pME-mNeongreen was used to generate the Hsa.TDP-43 Q331K construct. To study heterotypic condensates, a DNA construct was generated recombining pTol2-Myl7:FusionRed as destination vector with p5E-3mnx1 (44), pME-mScarlet3 and p3E-Hsa.TDP-43 G294V or delta NLS. All constructs were validated by sanger-sequencing through Australian Genome Research Facility. Wildtype TAB and transgenic line *Tg*(-*3mnx1*:Hsa.H2B-mCerulean3) were used for microinjections of pTol2-3mnx1: eGFP-Hsa.TDP-43 wildtype and mutant DNA plasmid.

#### mRNA generation

To generate mRNA, the plasmid pTol2pA2-3mnx1(GRCz11)-T7:eGFP-TDP-43^WT^ was generated as described in (10). mRNA was generated as described in (46) using mMESSAGE mMACHINE® T7 Transcription Kit (ThermoFisher #AM1345) and mRNA was cleaned up using MEGAclear Transcription (mRNA) Cleanup Kit (ThermoFisher #AM1908); both steps were performed according to the manufacturer's protocol. Concentrations were determined using Nanodrop and mRNA was microinjected (injection mix with 75 ng/μl mRNA) using droplets with diameters of 100, 150 and 200 microns corresponding to droplet volumes (amount) of 0.52 nl (0.039 ng RNA), 1.77 nl (0.132 ng RNA) and 4.19 nl (0.314 ng RNA) into the zebrafish zygote of transgenic fish *Tg(-3mnx1:mTagBFP)* at the one-cell stage of development.

#### Protein extraction and western blot analysis

Zebrafish embryos (3–4 days post fertilization) of the wild type and transgenic fish lines: *Tg(-3mnx1:eGFP-Hsa.TDP-43^WT^), Tg(-3mnx1:eGFP-Hsa.TDP-43^G294V^), Tg(-3mnx1:eGFP-Hsa.TDP-43^dNLS^) and Tg(-3mnx1:eGFP-Hsa.TDP-43^2KQ^)* were euthanized and collected (*n* = 13–30) and washed with Ringer Solution (58.2 mM NaCl 4.0 mM KCl 4.8 mM NaHCO_3_ pH 7) and PBS before lysis in RIPA buffer (50 mM Tris–HCl pH 7.4, 150 mM NaCl, 1 mM EDTA, 1% Triton-X-100, 1% NaH_2_PO_4_, 0.1% SDS) with complete EDTA-free Protease Inhibitor Cocktail and PhosSTOP™ Phosphatase Inhibitor Cocktail (Roche). Proteins were quantified by BCA protein assay, separated by 4–15% SDS-PAGE (BioRad), transferred and treated with a polyclonal rabbit anti-TDP-43 antibody (ProteinTech; 10782–8-AP; 1/10000) and a mouse monoclonal anti-GAPDH antibody (ProteinTech, 60004-1-Ig, 1/20 000) overnight at 4°C. Li-Cor secondary antibodies donkey anti-rabbit 680LT and donkey anti-mouse 800CW were used for revelation on Li-Cor Odyssey CLx (Li-Cor, LCR- 926-68023 and LCR-926-32212 respectively, 1/15000).

#### Live cell imaging of zebrafish motor neurons

Confocal microscopy of live larvae (3–5 dpf) embedded in 0.6%-1% low-melting point agarose on glass-bottomed dishes was performed using a Leica SP8 confocal microscope. Images were acquired as z-stacks of single motor neurons with a Leica 40×/1.10 HC PL APO CS2 water immersion objective. Time-lapse imaging to study the dynamics of biomolecular condensates was conducted on a Leica SP5 confocal microscope using 8 kHz Leica resonant scanner with a Leica 40x/0.80 HCX APO L U-V-I water immersion objective, imaging a z-stack of a whole motor neuron every 40 sec. Following laser lines for excitation of H2B-mCerulean3 and eGFP were used: 405, 488 nm. Multiple technical replicates were performed, and N-numbers are indicated.

#### Image analysis

Image analysis was performed in FIJI (1.53q) if not indicated otherwise. Size and fluorescence intensities of individual BMCs in zebrafish motor neurons and human cells were determined by measuring a ROI around individual BMCs on a single optical plane. Mean fluorescence intensity values of individual BMCs were *z*-score normalized between conditions. BMC count and BMC dynamics were performed in 3D using the spot detection and spot tracking function in Imaris (V9.7). The BMC volumes were analyzed using the 3D manager macro of the 3D suit in FIJI. Videos were created in Imaris (V9.7) and annotated with arrows using Clipchamp, a video-annotation program.

#### Nucleo-cytoplasmic fluorescence intensity analysis in 2D (plot profile)

Cytoplasmic and nuclear fluorescence intensities (as fluorescent grey values) were measured as plot profiles on a single optical plane along a line drawn through the whole diameter of individual motor neurons in FIJI. Minimum (Fmin) and maximum (Fmax) fluorescence intensity values were extracted separately for the nucleus and the cytoplasm. Fluorescence intensity values were z-score normalized for statistical analysis.

#### Nucleo-cytoplasmic fluorescence intensity analysis in 3D (FIJI)

3D fluorescence intensity analysis of nuclear and cytoplasmic hTDP-43 was performed as described in Svahn *et al.* (10) with the change of using the NucleusJ macro (freely available on GitHub) for automatic detection (thresholding) of the nucleus in 3D.

#### Fluorescence recovery after photobleaching (FRAP) analysis and quantification in zebrafish

FRAP experiments were performed on a Leica SP5 confocal microscope with a Leica 40x/0.80 HCX APO L U-V-I water immersion objective. Photobleaching of eGFP-Hsa.TDP-43 was conducted on a single nuclear droplet with a square bleach ROI of 0.36 μm^2^ by a 405 nm laser beam at 60–70% intensity with 6 bleach pulses (1.2 sec each) to achieve >60% bleaching of the fluorescence in the ROI. Fluorescence recovery was monitored with an argon laser (excitation: 488 nm) at 8% laser intensity for a total time of 415 sec at 5 sec intervals. The FRAP data was quantified using FIJI. The fluorescent intensities for the bleached droplet (BL), reference region (REF, whole cell) and background region (BG, outside the cell) were measured. Background values were subtracted from bleach and reference values to obtain corrected values and corrected bleach to corrected reference values were normalized (BL-BG/REF-BG). The corrected normalized values were then normalized to the average pre-bleach intensities and plotted as normalized fluorescence intensities against time. Curve fitting of normalized data was performed using the program-based tool, EasyFRAP (47) using a double normalization and two-component exponential equation. Calculated mobile fraction, t-half time to maximum recovery and *R*^2^ values (goodness of fit) were extracted. Only FRAP experiments with a bleach efficacy > 60%, *R*^2^ > 0.7 and gap ratio >0.6 were used for calculating the mean of normalized FRAP experiments per condition and curve fitting. FRAP experiments were conducted on only one droplet per motor neuron. The number of total FRAP experiments per condition and number of embryos is indicated in the figure legends. All data presented was conducted in three or more independent experiments.

#### Swimming assay

Swimming assays were performed as previously reported (48). Briefly, WT or transgenic fish lines expressing eGFP-TDP-43 WT or 2KQ (all on TAB background), were set up for breeding and each clutch was considered as *n* = 1. Equal number of positive fish were placed in a dish in an incubator (raised in a light–dark cycle) and at 6dpf, individual fish for each condition were placed each in its own well in a 48-well plate with 500 μl of E3 water. The Zebrabox (Viewpoint Behaviour Technology) was used to track the swimming behavior of each individual fish. The tracking protocol was in total 22 min long with the first two minutes regarded as baseline recording (control) followed by two alternating light and dark cycles of 5 min each at 100% target power at sensitivity level of 22. The total swimming distance for light and dark period and the total time swam was recorded and quantified.

### *In vitro* experiments

#### Cell line maintenance, DNA constructs and transfection

U2OS cells were passaged by first washing in 1× PBS 0.5 mM EDTA, followed by incubation for 5 min in trypsin-EDTA dissociation solution, prior to plating. Plasmids containing C-terminally EYFP-tagged TDP-43 variants (TDP-43-WT = Addgene#84911, TDP-43–5FL = Addgene#84914) and empty vector EYFP (Addgene#84910) for mammalian expression were a kind gift from Dr Aaron Gitler (49). U2OS cells were transfected using ViaFect reagent (Promega) according to the manufacturer's instructions.

#### Microscopy of cultured cells

Following transfection, cells were cultured for 48 h prior to any experimentation. For the determination of BMC number, size, fluorescence intensity, and anisotropy, cells were fixed for 20 min in pre-warmed 4% PFA in 1× PBS. Following fixation, cells were washed with 100 mM Tris (pH 8.0) and then permeabilized in 1× TBS with 0.2% Triton X-100 (v/v) for 5 min. After permeabilization, cells were counterstained with Hoechst 33342 at a 1:5000 dilution in TBS for 5 min. Cells were then washed 3 times in TBS and imaged immediately or stored for no longer than 48 h prior to imaging. Fixed cells were imaged on a Leica DMi8 widefield fluorescent microscope using either a 20× (NA 0.4) or 40× (NA 1.3) oil immersion objective. For oil immersion imaging, media on cells was replaced with 20 mM Tris (pH 8.0) 90% glycerol (v/v) for matching of refractive index.

#### FRAP of cultured cells

Fluorescence recovery after photobleaching (FRAP) of human cells was carried out using a Leica SP5 scanning confocal microscope using the FRAP wizard. Cells were maintained at 5% CO_2_ in a humidified chamber at 37°C throughout FRAP experiments. Cells were imaged using a 63× water immersion objective (NA 1.2). The argon laser was set to 80% power for all steps in FRAP. Image size was set to 256 × 256 pixels and a scan speed of 700 Hz was used in bi-directional scanning mode. Bleach settings were ‘use zoom in’ and ‘set background to 0′ with 100% transmission of laser at 488 nm. The time series consisted of 10 prebleach frames (2% 488 nm transmission), followed by 10 bleach frames (100% 488 nm transmission), and 184 post-bleach frames (2% 488 nm transmission), with a total duration of 96 seconds. FRAP analysis was carried out using FIJI where the fluorescence intensity in the bleached region, whole cell, and background were measured using the ROI manager. The ‘easyFRAP-web’ online tool (47) was used to perform curve fitting analysis. Data within the tool were double-normalized and fit with single exponential fits to the recovery curves.

### Single-molecule tracking (SMT)

#### SMT sample preparation

Single-molecule tracking was performed as described in McCann *et al.* (50). HEK_293 and HeLa cells were seeded at a density of 20 000 cells/well in 8-well chamber glass slides (Ibidi) coated with 0.5% gelatine 24 h prior to transfection. 200 ng of plasmid DNA/well of either Halo-tagged TDP-43 or mutant TDP-43 was transiently transfected into the cells using X-tremeGENE 9 Transfection Reagent kit (Roche, Basel, Switzerland). HEK-293 cells were incubated at 37°C with 5% CO_2_ for 24 h prior to imaging and HeLa cells were incubated at 37°C with 5% CO_2_ for 24 h then washed three times and then incubated in DMEM media for another 48 h prior to imaging. 45 min prior to imaging 1nM of JF549 Halo-tag dye was added directly to the media and cells were incubated for 10 min at 37°C with 5% of CO_2_. Following incubation, cells were washed twice 15 min apart and replaced with Fluorobrite DMEM (Glibco).

#### SMT acquisition

Images were acquired on a Nikon TIRF microscope at a TIRF angle of 61° to achieve HILO illumination. Samples were recorded with an iXon Ultra 888 EMCCD camera, filter cube TRF49909-ET-561 laser bandpass filter and 100× oil 1.49 NA TIRF objective. Cells were imaged using a 561nm excitation laser at a power density of 10.3 μW to perform two different acquisition techniques. A fast frame rate which uses a 50 Hz (20 ms acquisition speed) to acquire 6000 frames without intervals to measure displacement distribution and fraction bound, and a slow frame rate which uses a 2 Hz (500 ms acquisition speed) to acquire 500 frames without intervals to measure residence times.

#### SMT analysis

Masking of the nucleus was performed in ImageJ for all files (fast and slow tracking). For HeLa cell experiments molecules were identified and tracked using a custom-written MATLAB implementation of the MTT algorithm (51), known as SLIMfast (52). For experiments using Hek-cells molecules were identified and racked using SPT LocAndTrack (53). Parameters used for fast frame rate analysis: localization error: 10^−6.5^; Blinking (frames): 1; max # of competitors: 3; max expected diffusion coefficient (μm^2^/sec): timepoints = 5, clip factor = 4. Cells with <500 trajectories based on the above parameters were excluded from analysis. All trajectories with <7 (timepoints) tracks were removed from further analysis. The first four frames of each trajectory were used to calculate the mean squared displacement. Diffusion coefficient was calculated from each trajectory's mean squared displacement and plotted. An inflection point was determined on WT TDP-43 diffusion profile and used as a boundary to determine mobile vs immobile state for all conditions. To calculate mobile vs immobile state the # of trajectories in a state were divided by the total # of trajectories. SpotOn, a model-based analysis of single-molecule displacement, was used to determine diffusion coefficients by displacement and population fractions for two-state and three-state kinetic model (54). Parameters used for slow frame rate analysis: Localization error: 10^−7^; Blinking (frames): 1; max # of competitors: 3; max expected diffusion coefficient (μm^2^/sec): 0.1. Slow-tracking analysis was performed using MATLAB code written by Chen *et al.* (Calculatelength 2fitting V3) (55).

### 3D modeling of hTDP-43 2KQ and 4FL structure in complex with UG-rich RNA

A structural model of hTDP-43 2KQ and 4FL fragments (102–269) containing both RRM 1 & 2 binding to UG-rich RNA (reference PBD: 4BS2) was created using Phyre2 (http://www.sbg.bio.ic.ac.uk/∼phyre2/html/page.cgi?id=index). These 3D models were then compared to the NMR 3D structure of hTDP-43 WT when also in complex with UG-rich RNA (reference PBD: 4BS2) using Mol*viewer (https://molstar.org/viewer/).

### Quantification and statistical analysis

All *in vivo* data was randomized for analysis. GraphPad PRISM 9.1 (GraphPad Software, Inc.) was used to analyze data for significance and graph creation. Statistical differences were evaluated using two-tailed *t*-test/Mann–Whitney test (comparing two groups), one-way ANOVA/Kruskal–Walli's test (comparing three and more groups) after checking for normal (gaussian) distribution (Shapiro–Wilk normality test). ANOVA with Dunnett multiple comparison correction was used to evaluate the statistical significance for single molecule tracking data. If not otherwise indicated, individual data values are represented as mean with error bars showing the standard error of mean (SEM).

## Results

### Subcellular characterization of different variants of human TDP-43 in motor neurons *in vivo*

**Figure 1.**
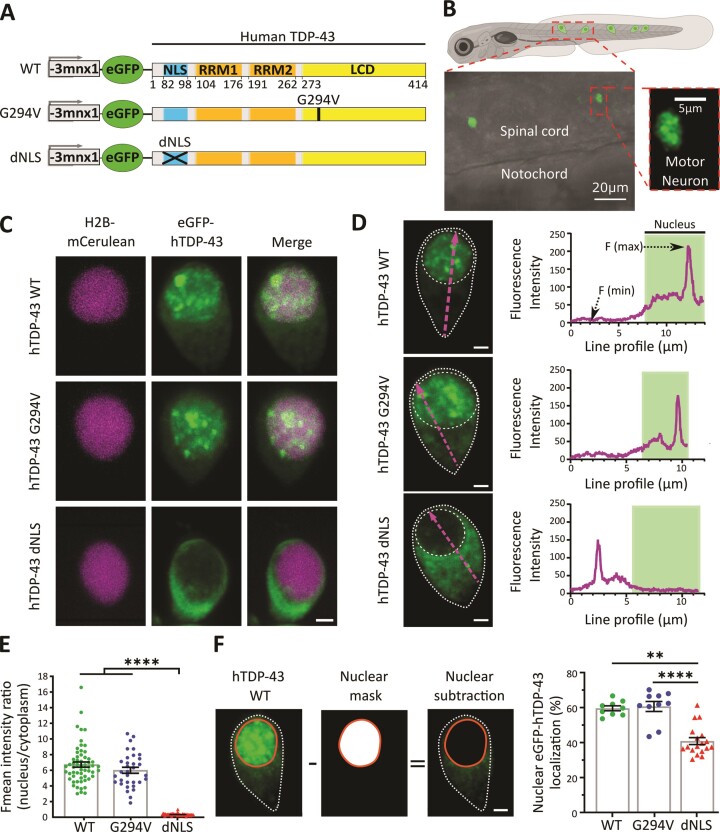
*In vivo* visualization of human TDP-43 in zebrafish spinal cord motor neurons. (**A**) Schematic of DNA constructs encoding for human wildtype TDP-43 (WT), an ALS mutant (p.G294V) and a nuclear localization signal (NLS) depleted version (dNLS), all with N-terminal tagged eGFP fluorophore under a neuron specific promoter (*-3mnx1*). (**B**) Representative confocal microscopy image of 3-day post fertilization old zebrafish expressing eGFP-hTDP-43 WT mosaically in spinal cord motor neurons. (**C**) Representative images of single motor neurons *in vivo* expressing the nuclear marker H2B-mCerulean3 (magenta) and eGFP-hTDP-43 WT, G294V or dNLS (green). (**D**) Fluorescence intensity measurements of eGFP-hTDP-43 along the cell axis (magenta dotted line) on single planes in individual motor neurons. Corresponding intensity plot profiles (right panels) illustrating compartment-specific eGFP-hTDP-43 expression. White dotted lines indicate the outline of the cell and nucleus. (**E**) Quantitative comparison of the mean fluorescence (Fmean) intensity ratios of nuclear to cytoplasmic plot profile measurements comparing hTDP-43 dNLS (0.34 ± 0.02) and G294V (5.99 ± 0.38) to WT (6.72 ± 0.33), (*n* = 58, 33, 57 motor neurons for WT, G294V, dNLS respectively); unpaired one-way ANOVA (****P ≤ 0.0001). (**F**) 3D analysis of eGFP-hTDP-43 compartment specific localization in cells co-expressing nuclear H2B-mCerulean3 showed 59.6% ± 1.4, 60.6% ± 2.9 and 37.6% ± 1.1 nuclear localization for hTDP-43 WT, G294V and dNLS respectively (*n* = 9, 10, 15 motor neurons for WT, G294V, dNLS); unpaired One-way ANOVA (**P = 0.0021, ****P ≤ 0.0001). Data points shown are mean ± SEM. Scale bars represent 2 μm.

To explore the sub-cellular distribution patterns of TDP-43, we expressed and analyzed different variants of human TDP-43 (Hsa.TDP-43, from here on referred to as hTDP-43) in caudal primary motor neurons in zebrafish. We generated DNA plasmids expressing either the full-length wild-type form of hTDP-43 (hTDP-43 WT), the familial/sporadic mutation hTDP-43 G294V, or cytoplasmic mislocalized hTDP-43 caused by a deletion of the nuclear localization signal (dNLS) (Figure 1A). All transgene variants were expressed using a neuron specific promoter (*-3mnx1*) and N-terminal tagged with enhanced GFP (eGFP). Injections resulted in mosaic expression of hTDP-43 in motor neurons along the spinal cord (Figure 1B). Construct expression was validated by immunoblot analysis (Supplementary Figure S1A). High-resolution confocal microscopy revealed that hTDP-43 WT showed a predominantly nuclear expression, as previously demonstrated (10). hTDP-43 G294V showed a similar expression pattern with dominant nuclear expression. As expected, depletion of the NLS resulted in predominant cytoplasmic localization (Figure 1C). To quantitatively determine the distribution of hTDP-43 expression in single motor neurons, we first assessed nuclear and cytoplasmic fluorescence intensities along the cell axis on single optical confocal sections (Figure 1D, magenta dashed lines). Notably, the plot profiles revealed distinct hTDP-43-eGFP accumulations in the nucleus and cytoplasm for hTDP-43 WT, G294V and dNLS respectively (see corresponding plot profiles in Figure 1D). The plot profiles allowed quantitative, compartment-specific (nucleus versus cytoplasm) fluorescence intensity readouts and confirmed predominant nuclear expression for hTDP-43 WT and G294V (Figure 1E; Supplementary Figure S1B–F). As expected, hTDP-43 dNLS showed a predominant cytoplasmic expression (Figure 1E; Supplementary Figure S1B–F). We next confirmed the compartment-specific TDP-43 distribution using 3D volume analysis (10) of whole motor neurons co-expressing the nuclear marker H2B-mCerulean3 (see Material and methods). This analysis revealed 60% nuclear localization for hTDP-43 WT and 61% for hTDP-43 G294V (Figure 1F). A notable fraction of eGFP-hTDP-43 (38%) in the dNLS variant colocalized with the nuclear marker albeit displaying a markedly lower fluorescence intensity (Supplementary Figure S1C, G).

Altogether, we demonstrate a predominant but not exclusive cytoplasmic distribution of hTDP-43 for the dNLS variant, while WT and G294V expressions were predominantly nuclear with no significant differences in their sub-cellular distributions.

### hTDP-43 phase separates into biomolecular condensates in spinal motor neurons

**Figure 2.**
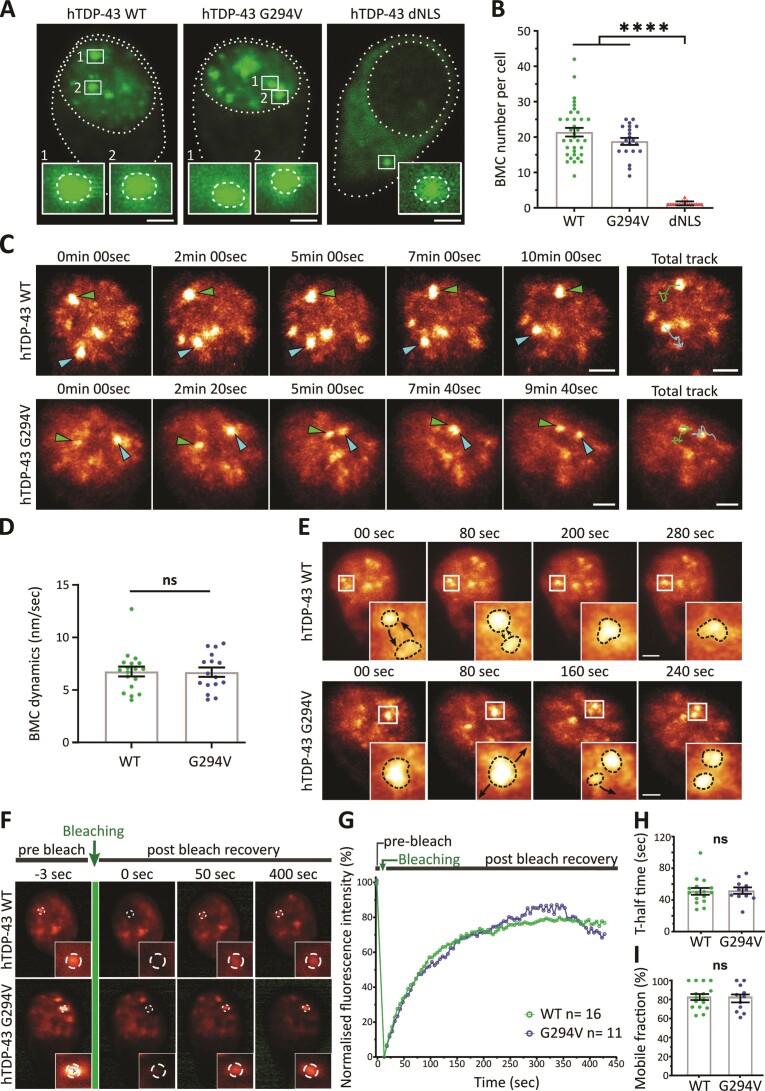
Quantitative characterization of hTDP-43 undergoing phase separation in zebrafish motor neurons *in vivo*. (**A**) Representative images of eGFP-hTDP-43 condensates in zebrafish motor neurons *in vivo* (nuclear for hTDP-43 WT and G294V, cytoplasmic for dNLS). (**B**) Quantification of nuclear BMC number per cell for WT (21.4 ± 1.2) and G294V (18.8 ± 1) and cytoplasmic BMCs for the dNLS mutant (1.2 ± 0.1), (*n* = 35, 21, 19 motor neurons for WT, G294V, dNLS); unpaired One-way ANOVA (****P ≤ 0.0001). (**C**) High-resolution time-lapse imaging revealed that BMCs are dynamic within the nucleus. Time-lapse videos located in Supplementary resource. (**D**) BMC dynamics were unaltered between hTDP-43 WT (6.8 ± 0.5 nm/sec) and G294V (6.7 ± 0.4 nm/sec) (*n* = 18, 16 BMCs from 6, 6 motor neurons for WT and G294V respectively); Mann–Whitney *t*-test (ns = non-significant). (**E**) Examples of fusion and fission events for hTDP-43 WT and G294V BMCs. Time-lapse videos located in Supplementary resource. (**F**) Representative images of the fluorescence recovery after photobleaching (FRAP) workflow of single nuclear BMCs in zebrafish spinal motor neurons (pre- and 400 sec post-bleach for hTDP-43 WT and G294V). (**G**) Mean fluorescence recovery curves normalized for background and photobleaching for hTDP-43 WT and G294V (*n* = 16, 11 cells from 12, 8 fish for WT and G294V respectively). (**H**) T-half time to maximum fluorescence recovery for hTDP-43 WT (50.9 sec ± 4.3) and G294V (51.8 sec ± 4.1); unpaired *t*-test (ns = non-significant). (**I**) Mobile fraction of BMCs expressing hTDP-43 WT (83% ± 3) and G294V (81% ± 4); unpaired *t*-test (ns = non-significant). Data points shown are mean ± SEM. Scale bars represent 2 μm.

*In vitro* reports have highlighted the importance of liquid-liquid phase separation (LLPS) for TDP-43, resulting in the formation of droplet-like structures or biomolecular condensates (BMCs) in order to carry out physiological cellular functions (5,9). In this *in vivo* model, hTDP-43 WT and G294V accumulated in multiple droplet-like structures inside the nucleus, whereas the hTDP-43 dNLS variant showed only occasionally bright, droplet-like structures in the cytoplasm (Figure 2A). To assess whether these nuclear hTDP-43 positive droplets present similar LLPS characteristics compared to *in vitro* studies, we quantitatively characterized the droplets *in vivo*. The overall number of droplets per cell (3D Imaris spot detection; see Material and methods) was not altered between hTDP-43 WT and G294V mutant but significantly lower in the dNLS version (Figure 2B). Measurement of the droplet areas (single optical plane) did not reveal a difference in size between hTDP-43 WT (0.16 μm^2^ ± 0.006), G294V (0.15 μm^2^ ± 0.006), and dNLS (0.15 μm^2^ ± 0.009) (Supplementary Figure S2A). This was confirmed by 3D volume analysis of individual droplets (Supplementary Figure S2B). Average fluorescent intensities of droplets were higher for hTDP-43 WT and G294V compared to the dNLS mutant (Supplementary Figure S2C). Phase separated condensates generally adopt a spherical shape due to their liquid properties, which was confirmed in zebrafish neurons (sphericity of 0.78 ± 0.009, 0.74 ± 0.012 and 0.72 ± 0.013 for WT, G294V and dNLS respectively; Supplementary Figure S2D). Cytoplasmic droplets were only occasionally observed in cells expressing hTDP-43 WT and G294V (11.9% and 3.5% respectively), with infrequent nuclear inclusions (17.6%) for the cytoplasmic dNLS variant (Supplementary Figure S2E).

Considering the low number of droplets in the dNLS mutant, we focused our characterization on the condensation properties of hTDP-43 WT and G294V in the nucleus. Time-lapse image acquisition of motor neuron somas (z-stack every 40–60 sec up to 15 mins) revealed that the nuclear droplets for hTDP-43 WT and G294V were dynamically moving within the nucleus (Figure 2C; Supplementary Videos S1 and S2). No difference in mean speed (or overall mean displacement) of individual droplets was observed between hTDP-43 WT and G294V (6.8 nm/sec versus 6.7 nm/sec; Figure 2D). Another characteristic of phase separated proteins is the ability to form membrane-less condensates that can undergo spontaneous fusion and fission. Consistent with this, we observed that *in vivo* droplets of hTDP-43 WT and G294V rapidly fused and separated over time (Figure 2E; Supplementary Videos S3 and S4). We occasionally observed that the same droplet underwent fusion and fission multiple times across a span of minutes.

An established approach to characterize phase separation properties and to assess protein mobility within BMCs is to measure the fluorescence recovery after photobleaching (FRAP) (56). We therefore performed FRAP on single hTDP-43 BMCs within the nucleus of an individual motor neuron in the living zebrafish spinal cord (Figure 2F). The fluorescent intensities were corrected for overall bleaching and normalized to the pre-bleach intensity (see Materials and methods, Supplementary Figure S3A, B). The recovery curves are (i) a valuable indicator of whether individual hTDP-43 molecules are mobile within BMCs (as expected for phase separated condensates) and (ii) allow quantification of such a molecular interchange (Figure 2G). A two-component exponential equation was confirmed to be the best fit to quantify the recovery curves using the software EasyFRAP (57) (Supplementary Figure S3C). Nuclear BMCs expressing hTDP-43 WT and G294V displayed a half-time to maximum fluorescence recovery (*t*_1/2_) of 50.9 sec (± 4.3) and 51.8 sec (± 4.1) respectively (Figure 2H). The mobile fraction (percentage of maximum fluorescence recovery) was unaltered with a percentage of 79% and 80% of their original pre-bleach fluorescence (Figure 2I). Considering the C-terminal domain of TDP-43 has several disordered motifs (22,58), we next performed FRAP analysis of TDP-43 Q331K (59) in zebrafish. No significant differences in fluorescence recovery were detected (Supplementary Figure S1H). To further evaluate whether FRAP dynamics might be perturbed as a result of different expression levels of hTDP-43, we performed a dose-response curve for hTDP-43 RNA injections. No variations in FRAP recoveries or nuclear/cytoplasmic distribution with increasing volumes of hTDP-43 RNA injections were observed (Supplementary Figure S1I-J).

To assess whether TDP-43 variants can colocalize into heterotypic BMCs in zebrafish motor neurons and whether this would affect condensate dynamics, we injected hTDP-43 G294V and dNLS (tagged with mScarlet3) into transgenic zebrafish expressing hTDP-43 WT (eGFP-tagged). TDP-43 G294V often formed heterotypic BMCs with a high degree of colocalization with WT hTDP-43 (Supplementary Figure S4A, B, Supplementary Table S1). As expected, cytoplasmic-targeted dNLS hTDP-43 did occasionally form heterotypic BMCs but showed less colocalization with WT hTDP-43 (Supplementary Figure S4A, B, Supplementary Table S1). FRAP analysis of the co-expressing condensates revealed unaltered t-half time across all groups, while heterotypic mScarlet3 hTDP-43 dNLS/eGFP-hTDP-43 WT condensates showed a decrease in their mobile fraction (Supplementary Figure S4C).

Overall, these results demonstrate that hTDP-43 variants phase separated into BMCs within the nucleus of zebrafish spinal cord motor neurons, displaying LLPS characteristics such as spherical shape, dynamic movement, ability to fuse and separate, and rapid fluorescence recovery after photobleaching.

### RNA-binding affects the cellular localization and LLPS properties of TDP-43 *in vivo*

**Figure 3.**
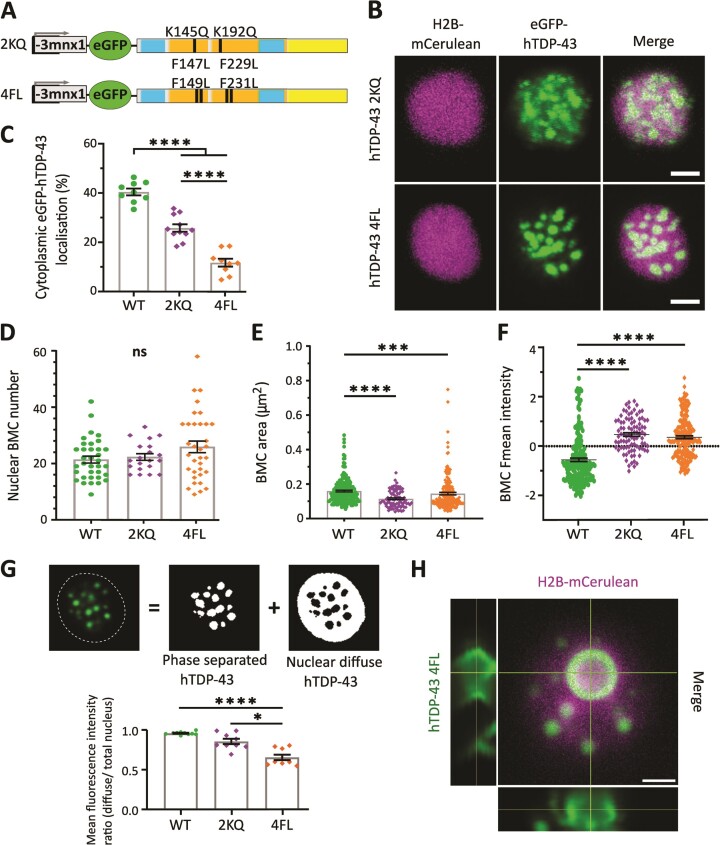
RNA binding deficiency affects compartmentalization and phase separation of hTDP-43 *in vivo*. (**A**) Schematic of DNA constructs encoding for RNA-binding deficient hTDP-43 2KQ and 4FL mutations with N-terminal tagged eGFP fluorophore under a neuron specific promoter (*-3mnx1*). (**B**) Representative images of motor neurons of 3 days post fertilization old embryos expressing the nuclear marker H2B-mCerulean3 (magenta) and eGFP-hTDP-43 2KQ and 4FL (green). (**C**) Quantification of 3D analysis of eGFP-hTDP-43 compartment specific localization in cells co-expressing H2B-mCerulean3 (cytoplasmic localization = 40.4% ± 1.4 for hTDP-43 WT, 25.8% ± 1.5 for 2KQ, and 11.7% ± 1.6 for 4FL; *n* = 9, 11 and 9 motor neurons for WT, 2KQ and 4FL respectively); unpaired one-way ANOVA (****p ≤ 0.0001). (**D**) Quantification of nuclear BMC number per cell for WT (21.4 ± 1.2), 2KQ (22.3 ± 1.2) and 4FL (25.88 ± 2), (*n* = 35, 20 and 34 motor neurons for WT, 2KQ and 4FL, respectively); unpaired one-way ANOVA (ns = non-significant). (**E**) Quantification of BMC area (WT = 0.159 μm^2^ ± 0.005; 2KQ = 0.115 μm^2^ ± 0.005; 4FL = 0.145 μm^2^± 0.007), (*n* = 204, 89, 189 BMCs of 62, 21 and 35 motor neurons for WT, 2KQ and 4FL respectively); unpaired One-way ANOVA (***P < 0.0002, ****P ≤ 0.0001). (**F**) *Z*-score normalized nuclear BMC mean fluorescence intensities for 2KQ (0.47 SD above the mean ± 0.07) and 4FL (0.36 SD above the mean ± 0.06) in comparison to WT (0.55 SD below the mean ± 0.07), (*n* = 204, 89, 189 BMCs of 62, 21 and 35 motor neurons for WT, 2KQ, 4FL, respectively); unpaired one-way ANOVA (****P ≤ 0.0001). (**G**) Analysis workflow quantifying the mean fluorescence intensity ratio of nuclear diffuse hTDP-43 to total nuclear hTDP-43 using a 3D segmentation macro (hTDP-43 WT = 0.96 ± 0.01; 2KQ = 0.86 ± 0.03; 4FL = 0.66 ± 0.03; *n* = 10, 9 and 9 cells for WT, 2KQ and 4FL respectively); unpaired one-way ANOVA (*P = 0.0297, ****P ≤ 0.0001). (**H**) Example image of the rare formation of an anisosome-like BMC for hTDP-43 4FL. Data points shown are mean ± SEM. Scale bars represent 2 μm.

*In vitro* reports have confirmed that the post-translational modification (PTM) acetylation of lysine residues can result in reduced RNA-binding of TDP-43 (5) and that this can affect the ability of TDP-43 to phase separate (5,9,36). We therefore investigated whether RNA-binding of hTDP-43 could influence the LLPS properties of nuclear BMCs *in vivo*. Two additional DNA constructs were created, one mimicking acetylation through the mutation of two lysines into glutamines (K145Q, K192Q; hereafter hTDP-43 2KQ), and another mimicking near-complete loss of RNA-binding capacity by replacing four phenylalanine residues with leucine (F147L, F149L, F229L, F231L; hereafter hTDP-43 4FL) in the two RNA recognition motifs (RRMs) (5) (Figure 3A). Modelling binding of the hTDP-43 RRM sites with UG-rich RNA (using the PDB model 4BS2 (26)) indicated the loss of a hydrogen bond (blue dotted line) between lysine 145 and guanine but not at lysine 192 in the 2KQ variant (Supplementary Figure S5A). Mutations of the leucine residues in the 4FL variant prevent pi-stack interaction between the phenyl group of the phenylalanine and the imidazole/pyrimidine group from the guanine of the RNA strand at positions 149 and 231 (Supplementary Figure S5B).

Expression of hTDP-43 4FL and 2KQ showed predominant nuclear expression with hTDP-43 phase separating into nuclear condensates *in vivo* (Figure 3B). 3D volume analysis and plot profile analysis revealed reduced cytoplasmic hTDP-43 localization for the RNA-binding deficient variants compared to hTDP-43 WT (11.7% for 4FL and 25.8% for 2KQ compared to 40.4% for WT) (Figure 3C; Supplementary Figure S6B–F). Nuclear droplet numbers per cell were consistent (Figure 3D) while the area of 2KQ and 4FL BMCs was significantly smaller (0.115 μm^2^ ± 0.005 and 0.145 μm^2^ ± 0.007 respectively) compared to WT (0.159 μm^2^ ± 0.005) (Figure 3E; Supplementary Figure S6G). BMCs maintained a spherical shape (Supplementary Figure S6H), and the normalized mean fluorescence of BMCs expressing hTDP-43 4FL and 2KQ was increased (0.47 SD above the mean ± 0.07 and 0.36 SD above the mean ± 0.06) compared to hTDP-43 WT (Figure 3F).

To determine the amount of diffuse nuclear hTDP-43 (not phase separated into BMCs) we performed 3D segmentation analysis (Figure 3G; see Materials and methods). The mean intensity ratios of diffuse nuclear hTDP-43 to total nuclear hTDP-43 revealed that the RNA-binding deficient mutant 4FL expressed significantly less diffuse hTDP-43 in the nucleoplasm (WT: 0.96, 2KQ: 0.86, 4FL: 0.66) (Figure 3G; also compare Figures 3B to 1C). No such changes were observed for the ALS-variant G294V (0.98; data not shown).

A recent *in vitro* study of RNA-binding deficient hTDP-43 (2KQ and 5FL) demonstrated a unique droplet formation with symmetrical liquid spherical shells and liquid cores, termed anisosomes (9). We generally observed *in vivo* a uniform distribution of eGFP-hTDP-43 throughout the whole BMC with a maximum fluorescence intensity in the center in all expressed variants (Supplementary Figure S6I, J). However, we did occasionally (∼5% of cells) observe enlarged BMCs in which eGFP-hTDP-43 4FL expression was concentrated in an outer shell (Figure 3H). These anisosome-resembling condensates generally appeared much larger in size (1.5–6.8 μm^2^ compared to an BMCs average of 0.17 μm^2^ in 4FL).

**Figure 4.**
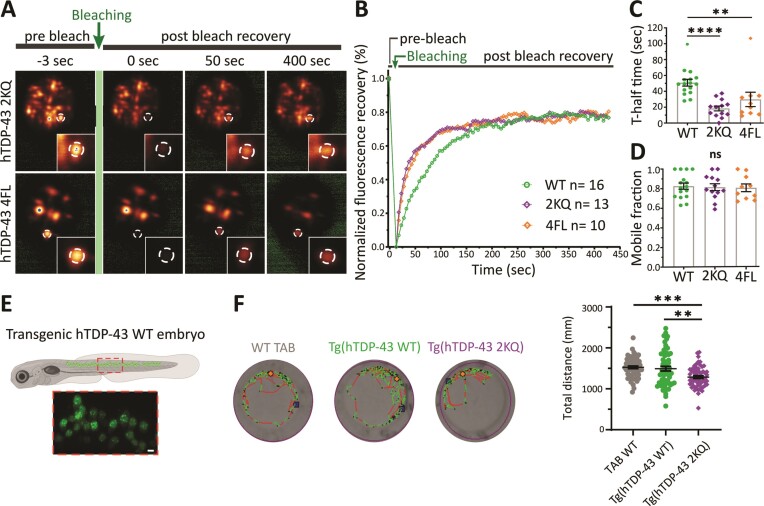
hTDP-43′s capacity to bind RNA influences its FRAP characteristics *in vivo*. (**A**) Representative images of the fluorescence recovery after photobleaching (FRAP) workflow of single nuclear BMCs in zebrafish spinal motor neurons (pre- and 400 sec post-bleach for hTDP-43 2KQ and 4FL). (**B**) Mean fluorescence recovery curves for hTDP-43 2KQ and 4FL normalized for background and photobleaching (*n* = 16, 13 and 10 cells from 12, 13 and 10 fish for WT, 2KQ and 4FL, respectively). (**C**) T-half time to maximum fluorescence recovery for hTDP-43 2KQ (18.4 sec ± 3.1) and 4FL (29.8 sec ± 9.1) are faster compared to WT (50.86 sec ± 4.3); unpaired one-way ANOVA (**P = 0.007, ***P ≤ 0.0001). (**D**) Mobile fraction of BMCs expressing hTDP-43 WT (83% ± 3), 2KQ (82% ± 4) and 4FL (81% ± 4); unpaired one-way ANOVA (ns = non-significant). (**E**) Schematic and representative confocal microscopy image of 3 days post fertilization old embryo from a stable transgenic line expressing hTDP-43 WT throughout the spinal cord. (**F**) Representative tracking images of one minute in the dark for WT TAB larvae and transgenic larvae expressing hTDP-43 WT or hTDP-43 2KQ. Quantification of the total distance travelled in the dark for WT TAB (1525 mm ± 31), hTDP-43 WT (1492 mm ± 57) and hTDP-43 2KQ (1286 mm ± 33) of three independent experiments (*n* = 61, 58, 61 embryos for WT TAB, hTDP-43 WT and hTDP-43 2KQ respectively); unpaired one-way ANOVA (**P = 0.0017, ***P = 0.0002). Data points shown are mean ± SEM.

We next analyzed the dynamics of RNA-binding deficient 2KQ and 4FL BMCs. Again, BMCs were mobile within the nucleus (Supplementary Figure S7A; Supplementary Videos S5 and S6) with the average speed of 2KQ BMCs (10.6 nm/sec) being significantly faster compared to hTDP-43 WT (6.8 nm/sec; Supplementary Figure S7B). BMCs for both RNA-binding deficient variants showed the ability to undergo fusion and separation events (Supplementary Figure S7C; Supplementary Videos S7 and S8). Importantly, FRAP analysis of RNA-binding deficient BMCs (Figure 4A) revealed that both the 2KQ and 4FL variants were able to recover after photobleaching (Figure 4B) and showed a significantly reduced half-recovery time (18.4 sec for 2KQ and 29.8 sec for 4FL compared to 50.9 sec for WT; Figure 4C). The mobile fractions were comparable to hTDP-43 WT (Figure 4D).

Lastly, to assess whether neuron-specific expression of an RNA-binding deficient variant can have a phenotypic effect, we generated transgenic fish expressing eGFP-hTDP-43 2KQ throughout the zebrafish spinal cord (Figure 4E) and assessed their swimming behavior in comparison to non-transgenic WT and eGFP-hTDP-43 WT lines. Notably, stable transgenic hTDP-43 2KQ zebrafish revealed a reduction in their swimming response after light stimuli compared to controls (Figure 4F).

Overall, these data revealed reduced cytoplasmic mislocalization of hTDP-43 2KQ and 4FL, with TDP-43 being concentrated into nuclear BMCs in zebrafish spinal motor neurons. Increased phase separation was further associated with a reduction in the levels of diffuse nuclear hTDP-43. hTDP-43 2KQ and 4FL showed faster kinetics (FRAP recoveries) and altered condensation characteristics, likely due to their RNA-binding deficiency and conceivably due to the reduced levels of diffuse TDP-43. These alterations were accompanied by a swimming deficit in the 2KQ transgenic zebrafish.

### Changed LLPS characteristic of RNA-binding deficient hTDP-43 *in vitro*

**Figure 5.**
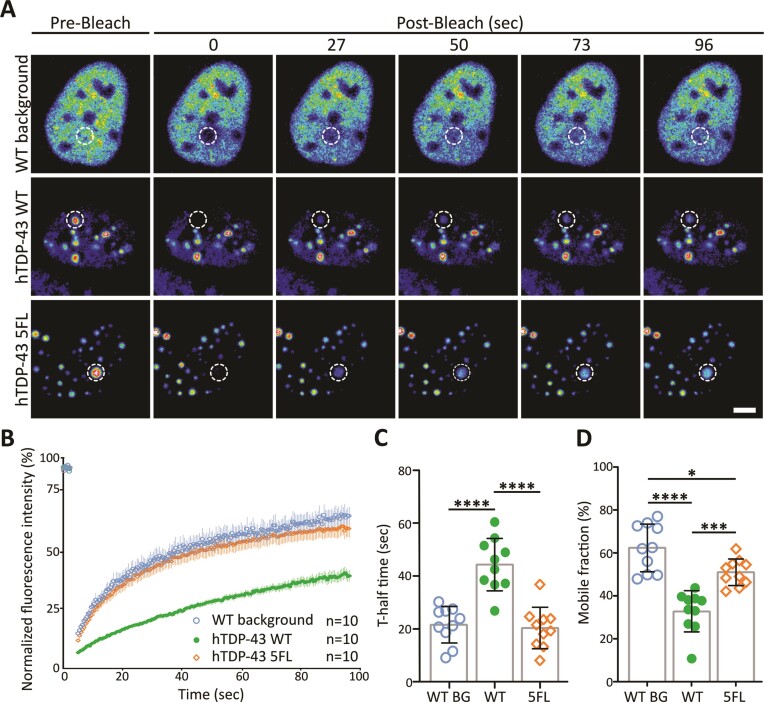
*In vitro* assessment of nuclear hTDP-43 5FL BMCs. (**A**) Representative images showing fluorescence recovery after photobleaching (FRAP) of non-phase separated nuclear diffuse hTDP-43 WT (WT background) and single BMCs expressing hTDP-43 WT and 5FL in U2OS cells. Scale bar 5 μm. (**B**) Normalized mean fluorescence recovery curves for WT background (blue circles), BMCs expressing hTDP-43 WT (green circles) and 5FL (orange diamonds). (**C**) Shorter *t*-half time to maximum fluorescence recovery for nuclear diffuse hTDP-43 WT (WT BG) (21.56 sec ± 2.19) and BMCs expressing hTDP-43 5FL (20.32 sec ± 2.49) compared to hTDP-43 WT (44.3 sec ± 3.14), (*n* = 10 BMCs for each condition); unpaired one-way ANOVA (****P ≤ 0.0001). (**D**) Higher mobile fraction of nuclear diffuse hTDP-43 WT BG (62.3% ± 3.5) and BMCs expressing hTDP-43 5FL (51% ± 2) compared to hTDP-43 WT BMCs (32.8% ± 3), (*n* = 10 BMCs for each condition); unpaired One-way ANOVA (*P < 0.05, ***P < 0.001, ****P ≤ 0.0001). Data points shown are mean ± SEM.

To further confirm that the inhibition of RNA-binding has a direct influence on hTDP-43 LLPS propensities, we transfected human bone osteosarcoma epithelial cells (U2OS; selected due to their favorable microscopy qualities) with human TDP-43 5FL, an RNA-binding deficient mutant with an additional phenylalanine to leucine mutation at amino acid 194 (9,25). Transfection of U2OS cells with the fluorophore alone (enhanced yellow fluorescent protein; eYFP) did not result in the formation of intranuclear BMCs (Supplementary Figure S8A). On the other hand, the expression of hTDP-43 WT and the 5FL mutant showed the formation of distinct intranuclear hTDP-43 condensates (Figure 5A and Supplementary Figure S8A). Fluorescence intensity measurements of single BMCs expressing hTDP-43 WT displayed a uniform distribution, similar to the *in vivo* observations (Supplementary Figure S8A, B). Notably, the RNA-binding deficient mutant 5FL revealed anisosome-like BMCs (Supplementary Figure S8A and B) (9). *In vitro* FRAP experiments on single intranuclear BMCs confirmed a significantly accelerated fluorescence recovery of hTDP-43 5FL compared to hTDP-43 WT BMCs (Figure 5B). The average recovery time (t-half time) was reduced by 54.1% compared to TDP-43 WT BMCs (Figure 5C). In these *in vitro* assays, the mobile fraction of 5FL (51%) was significantly greater than that of hTDP-43 WT (33%) (Figure 5D). Notably, the fluorescence recovery for hTDP-43 5FL BMCs was comparable to diffuse non-phase separated TDP-43 WT (Figure 5B, C). The droplet number and area were also significantly increased for hTDP-43 5FL compared to hTDP-43 WT, with no difference in overall mean fluorescence intensities of these condensates (Supplementary Figure S8C–E). Live-cell time-lapse microscopy further confirmed fusion and fission events of 5FL BMCs in this *in vitro* analysis (Supplementary Figure S8F). Altogether, the aberrant condensation characteristics observed in the RNA-binding impaired hTDP-43 *in vitro* assays were in line with the *in vivo* observations and highlight a role for these mutations and/or RNA-binding in regulating the molecular exchange of hTDP-43 within these condensates.

### Single-molecule tracking reveals altered population mobility and diffusion coefficients for hTDP-43 2KQ and 4FL

**Figure 6.**
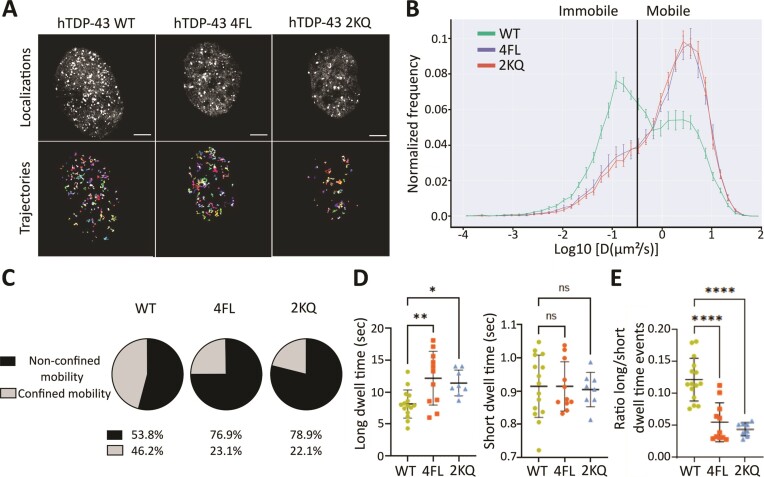
Single Molecule Tracking of hTDP-43 molecules in HEK cells showed an altered motility and diffusion profiles for RNA-binding deficient variants. (**A**) Visualization of localization and trajectories of single Halo-tagged hTDP-43 WT, 2KQ and 4FL molecules in the nucleus of HEK293 cells using HILO microscopy. Scale bar 5 μm. (**B**) Quantification of the mobility profile of HALO-hTDP-43 WT (green line), 2KQ (red line) and 4FL (blue line) using diffusion coefficient histogram (μm^2^/sec). Immobile and mobile fractions were classified using LogD = –0.5 as a threshold (*n* = 14, 15 and 13 for WT, 2KQ and 4FL respectively). (**C**) Pie charts quantifying the fractions of non-confined (mobile) and confined (immobile) mobility for wildtype hTDP-43, 2KQ and 4FL mutant. (**D**) Temporal binding dynamics of hTDP-43 molecules. Long dwell times (left graph) of the RNA-binding deficient mutants 2KQ (11.43 sec ± 2) and 4FL (12.26 sec ± 4) compared to WT (8.1 sec ± 2) and short dwell times (right graph) of hTDP-43 WT (0.9 sec ± 0.1), 2KQ (0.9 sec ± 0.05) and 4FL (0.9 sec ± 0.08), (*n* = 17, 8 and 13 cells for WT, 2KQ and 4FL respectively); ANOVA with Dunnett multiple comparison correction (ns = non-significant, *P < 0.05, **P < 0.01). (**E**) Ratio of event number of long to short dwell time events is reduced in 2KQ (0.04 ± 0.01) and 4FL (0.05 ± 0.03) mutants compared to WT (0.12 ± 0.03); (*n* = 17, 8 and 13 cells for WT, 2KQ and 4FL respectively); ANOVA with Dunnett multiple comparison correction (****P ≤ 0.0001). Data points shown are mean ± SD.

To investigate whether the RNA-binding deficient variants 4FL and 2KQ affect the molecular behavior of hTDP-43, we applied single-molecule tracking *in vitro* in HEK293 cells using Highly Inclined and Laminated Optical sheet (HILO) microscopy (60). This imaging approach, combined with sparse HALO labelling (see Materials and methods) enables the confident visualization and tracking of individual proteins within the nucleus (Figure 6A). Global analysis of the spatial mobility of the hTDP-43 variants 4FL and 2KQ revealed a significantly larger mobile population (78% and 76.1% respectively) compared to hTDP-43 WT (53.8%) (Figure 6B, C). Further analysis using either a 2-state or 3-state kinetic model of the jump-distance displacement showed a similar trend of RNA-binding deficient hTDP-43 revealing a larger mobile population (Supplementary Table S2).

Analysis of the temporal binding dynamics revealed two temporal populations, molecules that dwelled in the same location for either >1 sec (long dwell time) or <1 sec (short dwell time). The RNA-binding deficient mutants were found to have significantly increased long dwell times (11–12 sec) compared to hTDP-43 WT (8 sec), with no change of the short dwell times (Figure 6D). This indicates that the 4FL and 2KQ mutations significantly affect the interaction time of individual hTDP-43 proteins with each other and/or surrounding proteins and RNA. Quantifying the number of short and long dwell time events (ratio of long to short dwell time events) showed that RNA-binding deficient mutants 2KQ and 4FL had a significantly reduced long dwell time population in comparison to WT (Figure 6E). To confirm these results, we performed additional single-molecule tracking experiments in HeLa cells. Analysis revealed comparable molecular profiles, with hTDP-43 4FL revealing a larger mobile fraction and increased diffusion coefficients (Supplementary Figure S9).

Overall, the analysis of the molecular dynamics of nuclear hTDP-43 showed that RNA-binding deficiency resulted in a smaller population of hTDP-43 molecules that have a long interaction time (long dwell time) compared to WT, but when they interact, they do so for a longer period. Taken together, RNA-binding deficient hTDP-43 variants showed a fundamental change in their nuclear interactions (duration and number of events) with RNA, other proteins or chromatin, and therefore an altered molecular behavior *in vitro*.

## Discussion

In this study we demonstrate that TDP-43 fulfils critical criteria for liquid-liquid phase separation (LLPS) within motor neurons of living zebrafish. This includes the dynamic formation of spherical, membrane-less TDP-43 condensates in the nucleus of motor neurons, which can spontaneously fuse as well as undergo rapid molecular exchange with their surroundings. Importantly, we confirm that (i) nuclear TDP-43 undergoes aberrant phase separation *in vivo* as a result of RNA-binding deficiencies, and that (ii) a change in the molecular diffusion profile of TDP-43 *in vitro* well aligns with the altered phase separation behavior *in vivo*. These changes seem to be TDP-43 site-specific as the ALS-linked mutations G294V and Q331K in the low-complexity domain did not affect such phase separation behavior. Conversely, RNA-binding deficient TDP-43 showed aberrant condensation properties in the nucleus, which altered the abundance of diffuse TDP-43 in the nucleoplasm. Thus, we speculate that condensation of nuclear TDP-43 serves as a fundamental mechanism to regulate functional TDP-43. The importance of RNA-binding was further evidenced by altered FRAP recovery profiles, changes in single-molecule tracking of affected TDP-43 variants, as well as a swimming deficit in 2KQ transgenic zebrafish (summarized in Supplementary Table S3).

### Nuclear TDP-43 undergoes phase separation *in vivo*

ALS-associated RNA-binding proteins (RBPs) such as TDP-43 or FUS have been shown to undergo LLPS and form biomolecular condensates (BMCs) *in vitro* (5,6,8,9,61). Disease-linked mutations and post-translational modifications (PTMs) can change the structural and physicochemical properties of these RBPs and therefore influence their LLPS characteristics (6,61,62). However, most of the current *in vivo* work has been limited to the characterization of condensation snapshots at determined timepoints, i.e. analyzing stills of TDP-43 condensates before or after interventions (63–67). Nevertheless, Zhang *et al.* performed *in vivo* two photon imaging using a cranial window implantation procedure and followed the formation of TIA1 granules in the cortex of mice (63). Asakawa *et al.* performed *in vivo* imaging in zebrafish using optogenetic techniques with a focus on cytoplasmic aggregation (64). Challenges to assess the dynamic nature of condensation include their quick timeframes (seconds to minutes) and the need for high-resolution microscopy to observe these assemblies in sub-compartments of single cells over time. Zebrafish are optically translucent and provide a unique *in vivo* platform for such observations (68–70). Moreover, human proteins can be overexpressed in zebrafish neurons, and real-time imaging as well as cellular and molecular manipulations of proteins and condensates can be performed with relative ease (10,64). As such, here we provide a real-time and in-depth characterization of the dynamics of nuclear TDP-43 phase separation by assessing human ALS/FTLD-linked variants *in vivo* when expressed in zebrafish spinal motor neurons. We demonstrate, using time-lapse live imaging, that TDP-43 condensates move actively within the nucleus, undergo fusion and fission, and display rapid molecular exchange.

### ALS-linked mutations in the disordered domain did not alter TDP-43 LLPS properties

Most TDP-43 mutations found in ALS patients are located in the low-complexity domain (LCD), suggesting that disruption of this particular domain can contribute to disease pathology (20). A previous report found the ALS variant p.G294V to be pathogenic in an Australian cohort (71), which was subsequently confirmed in other cohorts (72–74). The functional effects of the G294V mutation are yet to be fully established. Our zebrafish observations did not display significantly altered compartment specific expression of hTDP-43 G294V compared to hTDP-43 WT (this study and (75)). These findings align with two other studies reporting unaltered cytoplasmic expression levels in primary patient fibroblasts (74) and iPSCs (76). There is some evidence though that mutations such as G294V can alter the amyloid properties of TDP-43 (77). Intriguingly, assessment of the molecular exchange of nuclear condensates also did not reveal significant alterations between WT, G294V and Q331K. Nevertheless, it is conceivable that alternative triggers (e.g. age) may lead to aggregate formation and thus contributing to pathogenesis (78,79). Further work will be required to determine the exact role of p.G294V and other mutations in nuclear condensation characteristics and in disease pathogenesis.

### RNA-binding deficiency of TDP-43 affects its LLPS properties *in vitro* and *in vivo*

The capacity of TDP-43 to bind hundreds of different RNAs makes it a crucial regulator for the life cycle of RNA (25,26,80). Modifications within the RNA-binding domains of TDP-43 severely impact its native protein folding properties, which have been predominantly shown to affect phase separation and ultimately cytoplasmic aggregation (5,8,36). However, two recent *in vitro* studies demonstrated the importance of condensation properties of TDP-43 in the nucleus as an important physiological concept (9,81). To assess the effect of RNA binding on nuclear condensation *in vivo* we tested an acetylation mimic variant (2KQ) and a variant with mutated RNA recognition sites (4FL). Both variants have been shown to result in drastically reduced RNA-binding of TDP-43 *in vitro*, and in our study both TDP-43 variants displayed increased nuclear retention and decreased cytoplasmic levels of hTDP-43. Indeed, several studies recently highlighted that nuclear retention might be independent of active nuclear export and that RNA binding and oligomerization play important roles in this process (82–86). Reduced RNA-binding capacity also resulted in faster FRAP recovery of nuclear BMCs and an overall higher nuclear BMC mobility (2KQ) *in vivo*. *In vitro* validation using the RNA-binding deficient variant 5FL (4FL with additional F194L mutation) confirmed an accelerated FRAP recovery. While our study did not directly investigate how TDP-43 function may be altered, phenotypic analysis of 2KQ expressing zebrafish revealed a swimming deficit, highlighting that motor-neuron restricted expression of RNA-binding deficient TDP-43 can have a functional impact. Nevertheless, we cannot exclude the impact that acetylation might have had in this setting (noting that this variant is an acetylation-mimic with a demonstrated RNA-binding deficit (5)). However, the similarities observed between 2KQ and 4FL in this study do support the hypothesis that RNA binding plays a central role in regulating condensation behavior *in vivo* and therefore ultimately contributes to TDP-43 accessibility in the nucleus.

### Single-molecule tracking reveals altered diffusion kinetics in RNA-binding deficient variants of TDP-43 *in vitro*

By applying single-molecule tracking *in vitro*, we determined that the molecular profile of RNA-binding deficient TDP-43 was significantly altered compared to TDP-43 WT. Both RNA-binding deficient TDP-43 variants (2KQ and 4FL) showed significantly increased diffusion coefficients as indicated by the larger mobile population of hTDP-43 molecules in the nucleus. These data conceivably explain a molecular underpinning for the surges in FRAP recovery of RNA-binding deficient hTDP-43 that were observed in this study and reported *in vitro*. Efficient TDP-43-RNA-interactions may help to stabilize TDP-43 in a functional sense but might limit the mobility of wild-type TDP-43. The data are also in line with a recent report from Streit *et al.*, in which single-molecule tracking revealed reduced mobility of wild-type TDP-43 within different compartments (nucleus, cytoplasm and stress granules) under stress conditions (40). Single-molecule tracking analysis performed here revealed that RNA-binding deficient TDP-43 variants exhibited prolonged temporal interactions (long dwell times), usually thought of as specific binding of proteins to chromatin. No difference in short dwell times, which are considered as scanning behavior for binding motifs or non-specific interaction with chromatin, was observed. In the case of TDP-43, these interactions may not be limited to chromatin and can include protein-RNA or protein-protein interactions. Very few studies have investigated the molecular interactions of TDP-43 with chromatin. One study identified loss of nuclear TDP-43 to be associated with chromatin decondensation, while another study reported a role for C9ORF72 and TDP-43 in chromatin remodeling and histone modifications (87,88).

In summary, RNA-binding deficient TDP-43 displayed enhanced diffusion coefficients while overall displaying fewer binding events. But in cases where binding occurred, the binding times (long dwell times) were increased. Steric or folding variations resulting from the introduced mutations in the RRM regions may make a ceasing of the interaction more difficult, leading to longer lived binding which may be interpreted as a regulatory mechanism of functional TDP-43 in the nucleus and/or a precursor for its transition from a liquid to a solid state (89). Although SMT and FRAP show some divergence in population behavior, this can be explained by the differences in modalities for image acquisition. SMT data are acquired at milli-second frame rates whereas FRAP data is acquired in sec/min. Of note, FRAP captures the collective response of a group of molecules that contribute to fluorescence recovery, while SMT detects a distribution of individual molecules behaviors within a specific population. Altogether, we speculate that a change in the molecular diffusion profile for 2KQ and 4FL TDP-43 might be an underpinning mechanism for aberrant LLPS. However, we cannot exclude secondary effects to RNA binding, such as structural or binding alterations due to the introduced mutations. 3D modelling of hTDP-43 certainly suggested some impact of 2KQ and 4FL mutations on RNA binding (Supplementary Figure S5) (5).

Overall, our data indicate that the 4FL and 2KQ mutations significantly affect the nuclear phase separation characteristics of TDP-43 and can retain TDP-43 in the nucleus *in vivo*. We hypothesize that this might have functional consequences of how individual TDP-43 proteins interact with themselves, surrounding proteins, or RNA. While our study did not directly assess these functional consequences, other studies have previously reported the influence of TDP-43 condensation on its function (59,90). Hallegger and colleagues used TDP-43 deletion constructs to reveal that the condensation propensities of TDP-43 tune RNA binding and that condensation is essential for its autoregulation (59). Schmidt *et al.* reported that certain C-terminal mutants displayed exon skipping defects but that overall IDR-driven condensation is dispensable for exon skipping (90). Further molecular studies into the binding interactions of TDP-43 will be crucial to better understand TDP-43 homeostasis and function in the nucleus, as well as to develop targeted interventions to prevent pathological aggregation in the cytoplasm (17,91).

### TDP-43 forms anisosomes *in vitro* and occasionally *in vivo*

**Figure 7.**
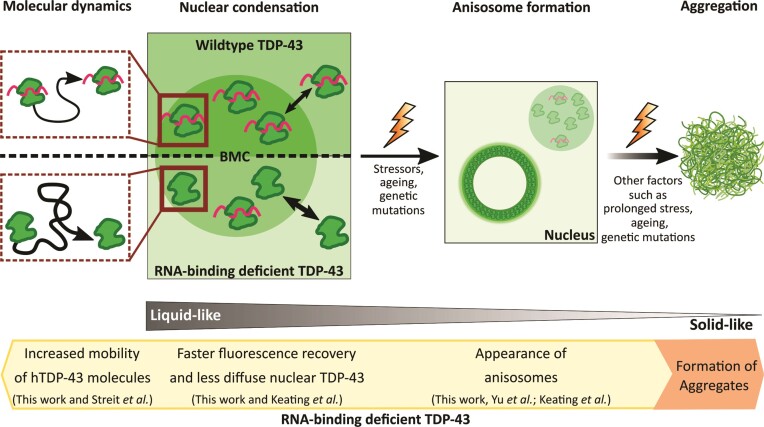
Schematic of the proposed transition from a liquid-to-solid state of RNA-binding deficient condensates. Depicted is a proposed mechanistic cascade of nuclear RNA-binding deficient TDP-43 condensates transitioning from liquid-liquid phase separated condensates to potential solid-like aggregates. Single-molecule tracking, and quantitative analysis used in this study showed an increased diffusion and mobility of single hTDP-43 molecules when mutations resulting in RNA-binding deficiencies were introduced. Moreover, nuclear biomolecular condensates (BMCs) displayed an increased motility, faster fluorescence recovery and a reduction in nuclear diffuse hTDP-43. BMCs expressing the 4FL mutation occasionally transitioned into anisosomes with TDP-43 concentrated in an outer shell *in vivo*. Thus, the molecular changes underlying this potentially stress-induced alteration of nuclear BMCs might represent a key step in the transition of condensates from liquid-like BMCs to anisosomes and ultimately irreversible solid-like aggregates.

A recent *in vitro* study observed that the RNA-binding deficient TDP-43 5FL (F147/149/194/231/233L) and 2KQ phase separated into multiphase condensates composed of a liquid core containing the heat shock protein 70 (HSP70) surrounded by a liquid shell with densely packed RNA-free TDP-43, termed anisosomes (9). The addition of an HSP70 inhibitor converted TDP-43 2KQ anisosomes into uniform droplets and decreased FRAP recovery of TDP-43 (9). Another study also showed that proteolytic impairment and arsenite treatment led to the anisotropic condensation of endogenous TDP-43 (81). We also observed the formation of anisosome-like condensates when expressing hTDP-43 5FL in human U2OS cells. In zebrafish spinal motor neurons, condensates generally showed a consistent fluorescence intensity profile. Nevertheless, we occasionally observed the formation of anisosome-like TDP-43 droplets in the 4FL RNA-binding deficient mutant. RNA-binding alterations might be a critical first step in this process, considering that high RNA concentrations in the nucleus are believed to retain BMCs in a liquid state (92). The comparably lower concentrations of RNA in the cytoplasm may also explain why we observed reduced condensation with our TDP-43 delta NLS constructs. RNA binding conceivably helps to stabilize TDP-43 in a functional state as decreasing the binding affinity of TDP-43 to RNA increases BMC formation and FRAP recovery. The introduction of oligonucleotides targeted to TDP-43 was further able to prevent aberrant phase separation and neurotoxic phase transition into aggregates in the cytoplasm (19,91). Notably, we (and another *in vitro* study) observed a reduction of free, diffuse nuclear TDP-43 with increased condensation in the nucleus (81). We therefore propose that nuclear phase separation of TDP-43 is most likely part of the physiological regulation of its own activity. And while LLPS is generally considered to facilitate physiological functions of TDP-43, increased condensation may also be able to limit the availability of otherwise functional wildtype TDP-43. Indeed, the conformational change of condensates into anisosomes accompanied by the clearance of functional, diffuse TDP-43 might represent a key step in the transformation from liquid-like into irreversible solid condensates (Figure 7). Further characterization of how condensation affects TDP-43 function in different compartments (e.g. nucleus versus cytoplasm versus stress granules) and under different conditions (e.g. physiological versus stress-exposed) will be critical to harvest its potential in drug discovery and development.

### Open questions and outlook

While these data provide *in vivo* demonstration of a potential molecular driver that can alter LLPS in the nucleus, many questions remain unanswered. For example, how does the overexpression of human protein in addition to endogenous protein affect condensation? In this study human TDP-43 analysis was performed in the presence of endogenous zebrafish TDP-43 and at modest overexpression levels. This might affect the condensation properties of human TDP-43, but may also mimic disease state, where mutations likely affect one copy of the gene resulting in heterozygous expression of the two variants. Another important question is how altered nuclear phase separation of TDP-43 contributes to loss of nuclear function, cytoplasmic mislocalization and aggregation? While the expression of two TDP-43 variants in our zebrafish did not alter FRAP recoveries, a recent study highlighted that TDP-43 co-expression can affect the abundance of diffuse TDP-43 levels and sequester endogenous TDP-43 (81). Other open questions surround the phase separation profiles of TDP-43 in anisotropic or cytoplasmic condensates? Or how do specific mutations, PTMs or even stressors alter the molecular structure of TDP-43 and therefore its phase separation properties? Future work using targeted approaches will be required to pinpoint the specific molecular mechanisms that can alter the biophysical properties of ALS-proteins in disease-relevant circumstances. Importantly, the *in vivo* model that we present in this study represents a key experimental platform to study LLPS characteristics as well as assessing the long-term effects of how ALS-linked TDP-43 mutations can affect neuron physiology. It will be interesting to see whether mutations or mislocalization of TDP-43 can trigger adult-onset alterations (e.g. accelerated motor neuron loss) or show increased susceptibility to internal or external stressors. This observational platform provides a unique opportunity for testing and confirming LLPS properties that have been previously identified *in vitro*, as well as extending this characterization to cytoplasmic condensates. Such *in vivo* validation will undoubtedly improve our understanding of factors that regulate RNA processing and TDP-43 homeostasis—helping to enhance the design of clinical interventions aiming to control and maintain TDP-43 function and solubility.

## Supplementary Material

gkae112_Supplemental_files

## Data Availability

Further information and requests for resources and reagents should be directed to the lead contact Marco Morsch (marco.morsch@mq.edu.au). U2OS cells were sourced from CellBank Australia (Cat No. 92022711). HEK293 cells were a gift from Professor Alpha Yap (The University of Queensland, QLD, Australia) and HeLa cells were a gift from Professor Geoffrey Faulkner (Translational Research Institute, Woolloongabba, QLD, Australia). Any inquiries for plasmids can be directed to the lead contact. This paper does not report original code. All data reported in this study will be shared by the lead contact upon request. Any additional information required to reanalyze the data reported in this paper is available from the lead contact upon request.
