# Supplementary material for: RNA-binding properties orchestrate TDP-43 homeostasis through condensate formation in vivo
Source: Nucleic Acids Res. 2024 Feb 21;52(9):5301–19. doi: 10.1093/nar/gkae112 (PMC11109982; doi:10.1093/nar/gkae112)
Supplement: gkae112_Supplemental_files [file gkae112_supplemental_files.zip › Supplementary Figures and Tables.pdf]

Supplementary Figures

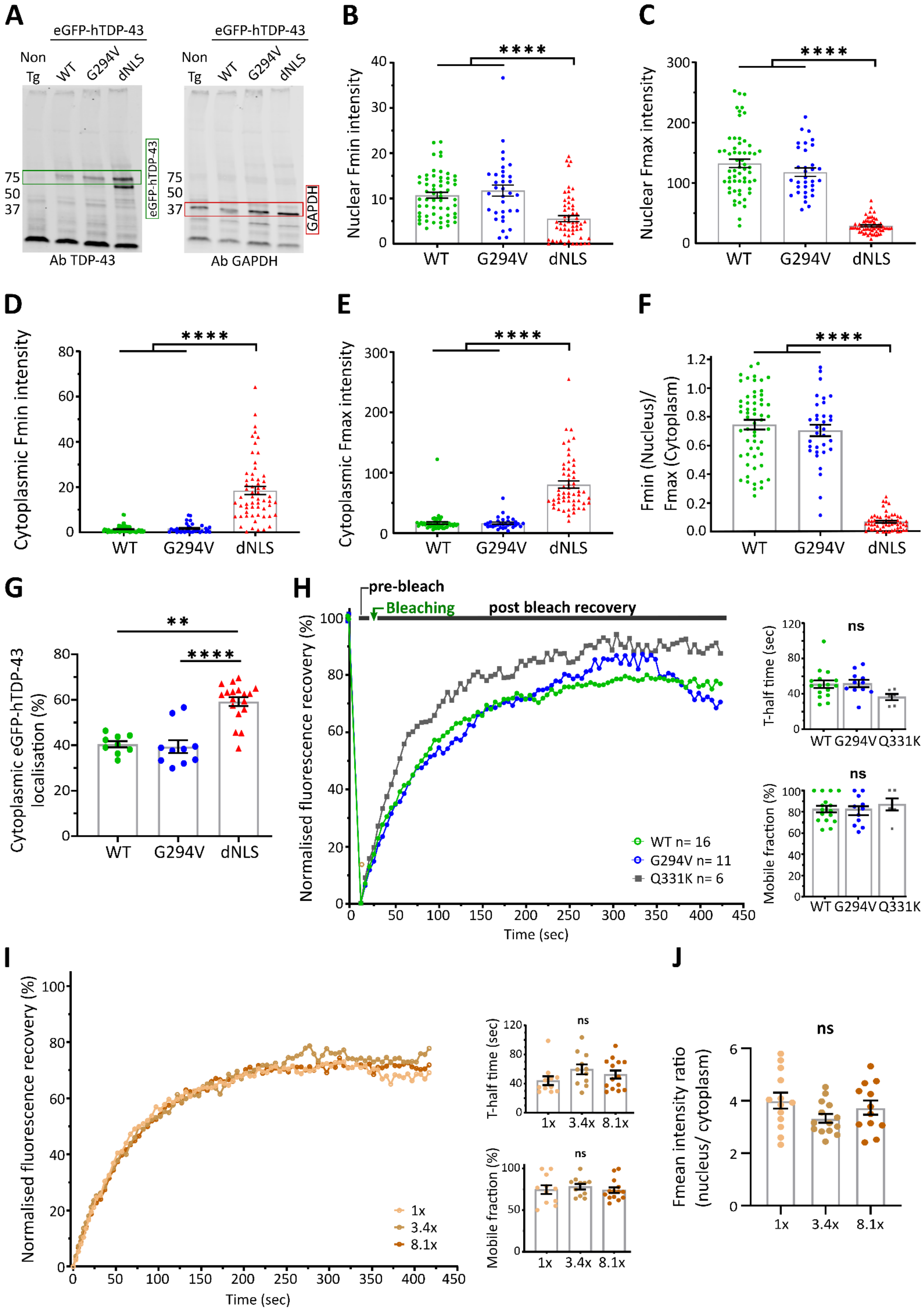

Supplementary Figure 1: Distribution of hTDP-43 WT and mutants in spinal motor neurons *in vivo*. (**A**) Western blot analysis of total protein extract of WT TAB embryos and transgenic embryos expressing eGFP-hTDP-43 WT, G294V or dNLS using antibodies against TDP-43 and GAPDH (n= 13-30 embryos at 3-4 dpf). (**B-E**) 2D fluorescence intensity measurements of eGFP-hTDP-43 variants along a line across the axis of single motor neurons showed (**B**) higher nuclear minimum fluorescence (Fmin) intensities and (**C**) higher nuclear maximum fluorescence (Fmax) intensities as well as (**D**) lower cytoplasmic Fmin intensities and (**E**) lower cytoplasmic Fmax intensities for hTDP-43 WT and G294V when compared to the dNLS variant (n= 59, 33 and 56 motor neurons for WT, G294V and dNLS respectively); unpaired One-way ANOVA (****p ≤ 0.0001). (**F**) The ratio of mean nuclear Fmin and mean cytoplasmic Fmax (2D plot profile measurement) confirmed the predominant nuclear expression for the hTDP-43 WT and G294V and the mostly cytoplasmic localization of the dNLS variant; unpaired One-way ANOVA (****p ≤ 0.0001). (**G**) 3D volume quantification of motor neurons co-expressing the nuclear H2BmCerulean3 marker confirmed the significant increase of cytoplasmic hTDP-43 dNLS (59.2% ± 2) compared to hTDP-43 WT (40.4% ± 1.4) and G294V mutant (39.4% ± 2.9) (n= 9, 10, 18 motor neurons for WT, G294V, dNLS respectively); unpaired One-way ANOVA (**p = 0.0021, ****p ≤ 0.0001). (**H**) Mean fluorescence recovery curves (normalized for background and photobleaching; same as Fig. 2G) for hTDP-43 WT, G294V and Q331K (n= 16, 11, 6 cells from 12, 8, 4 fish for WT, G294V, Q331K respectively). No differences in t-half time (Q331K: 36.1 sec ± 3.6) or mobile fraction (Q331K: 87% ± 6) were observed compared to WT or G294V; unpaired One-way ANOVA (ns=non-significant). (**I**) Normalized mean fluorescence recovery curves of BMCs when using different injection volumes of *hTARDPB WT* RNA: 0.52nl (1x), 1.77nL (3.4x) and 4.19nL (8.1x). No differences in t-half time (1x: 44 sec ± 6; 3.4x: 60 sec ± 7; 8.1x: 52 sec ± 6) or mobile fraction (1x: 75% ± 5; 3.4x: 78% ± 3; 8.1x: 74% ± 3) were observed, (n= 11, 11 and 14 cells from 4, 4 and 2 fish respectively); One-way ANOVA (ns = non-significant). (**J**) The mean fluorescence intensity (Fmean) ratio of nucleus and cytoplasm (2D plot profile measurements) did not show a significant difference when using different injection volumes of *hTARDBP WT* RNA; unpaired One-way ANOVA (ns=non-significant). Data points shown are mean ± SEM.

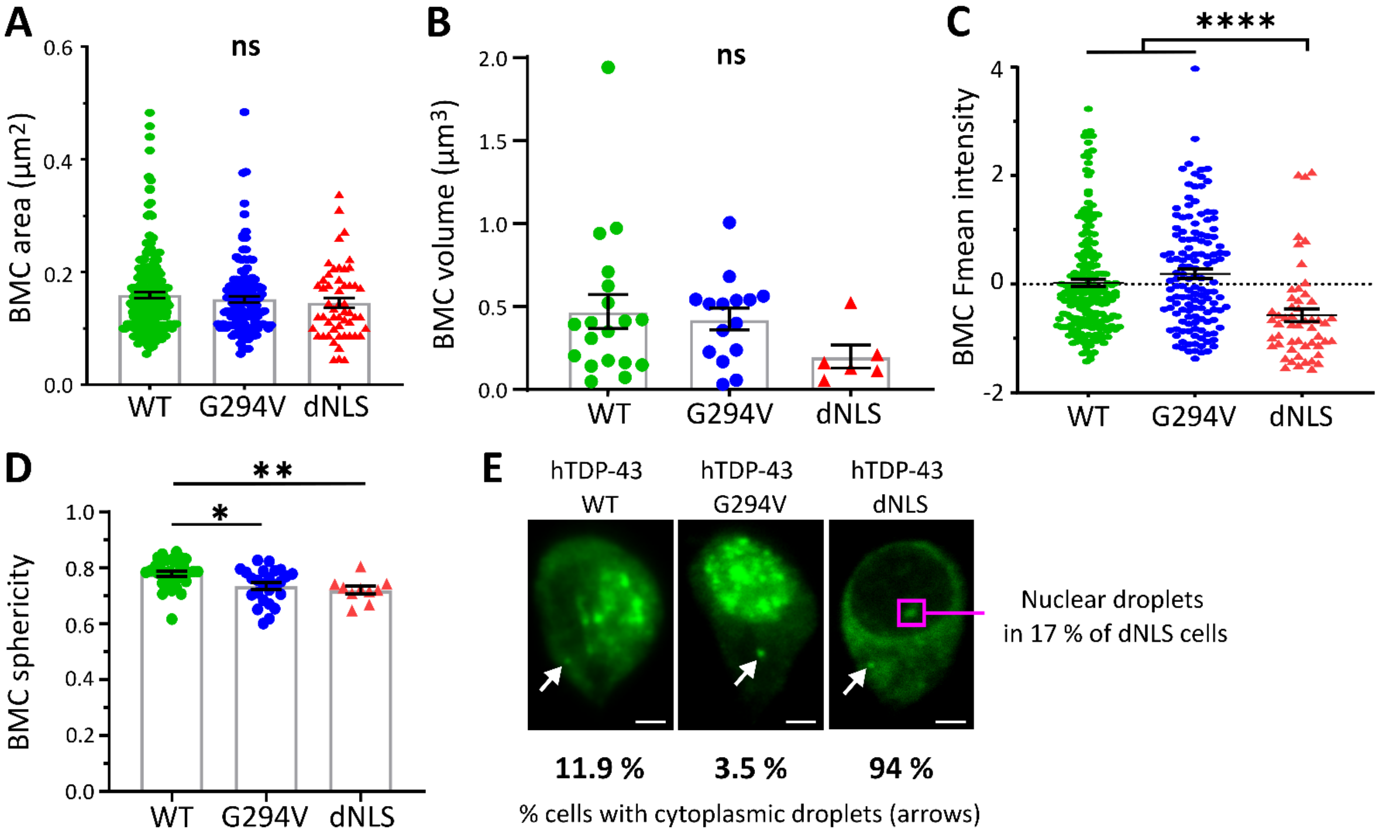

Supplementary Figure 2: Quantitative characterization of phase separated hTDP-43 WT, G294V and dNLS *in vivo.* (**A**) Quantification of BMC area showed no significant difference in size between hTDP-43 WT (0.159 µm^2 ± 0.006), G294V (0.152 µm^2 ± 0.006) and dNLS mutant (0.146 µm^2 ± 0.009) (n= 204, 134, 53 BMCs of 63, 46 and 31 motor neurons for WT, G294V, dNLS respectively); unpaired One-way ANOVA (ns=non-significant). (**B**) Quantification of BMC volume after 3D rendering of hTDP-43 WT (0.47 µm^3 ± 0.10), G294V (0.42 µm^3 ± 0.07) and dNLS mutant (0.20 µm^3 ± 0.07) showed no significant difference (n=19, 15, 6 BMCs for WT, G204V and dNLS respectively); unpaired One-way ANOVA (ns=non-significant). (**C**) Cytoplasmic hTDP-43 dNLS BMCs showed a decreased mean fluorescence (Fmean) intensity (0.57 SD below the mean ± 0.12) compared to WT (0.02 SD above the mean ± 0.07) and G294V (0.19 SD above the mean ± 0.09) after z-score normalization (n= 204, 134, 53 BMCs of 63, 46, 31 motor neurons for WT, G294V, dNLS respectively); unpaired One-way ANOVA (****p ≤ 0.0001). (**D**) Imaris 3D rendered BMCs expressing hTDP-43 WT (0.78 ± 0.009), G294V (0.74 ± 0.012) and dNLS (0.72 ± 0.013) were equally spherical in shape (n= 32, 25, 10 for WT, G294V and dNLS respectively); unpaired One-way ANOVA (*p < 0.217, **p < 0.0073). (**E**) In a small percentage of cells expressing hTDP-43 WT (11.9%) and G294V (3.5%) cytoplasmic condensates were observed, whereas 94% of all hTDP-43 dNLS cells had cytoplasmic condensates. Data points shown are mean ± SEM (exception C). Scale bar represents 2 µm.

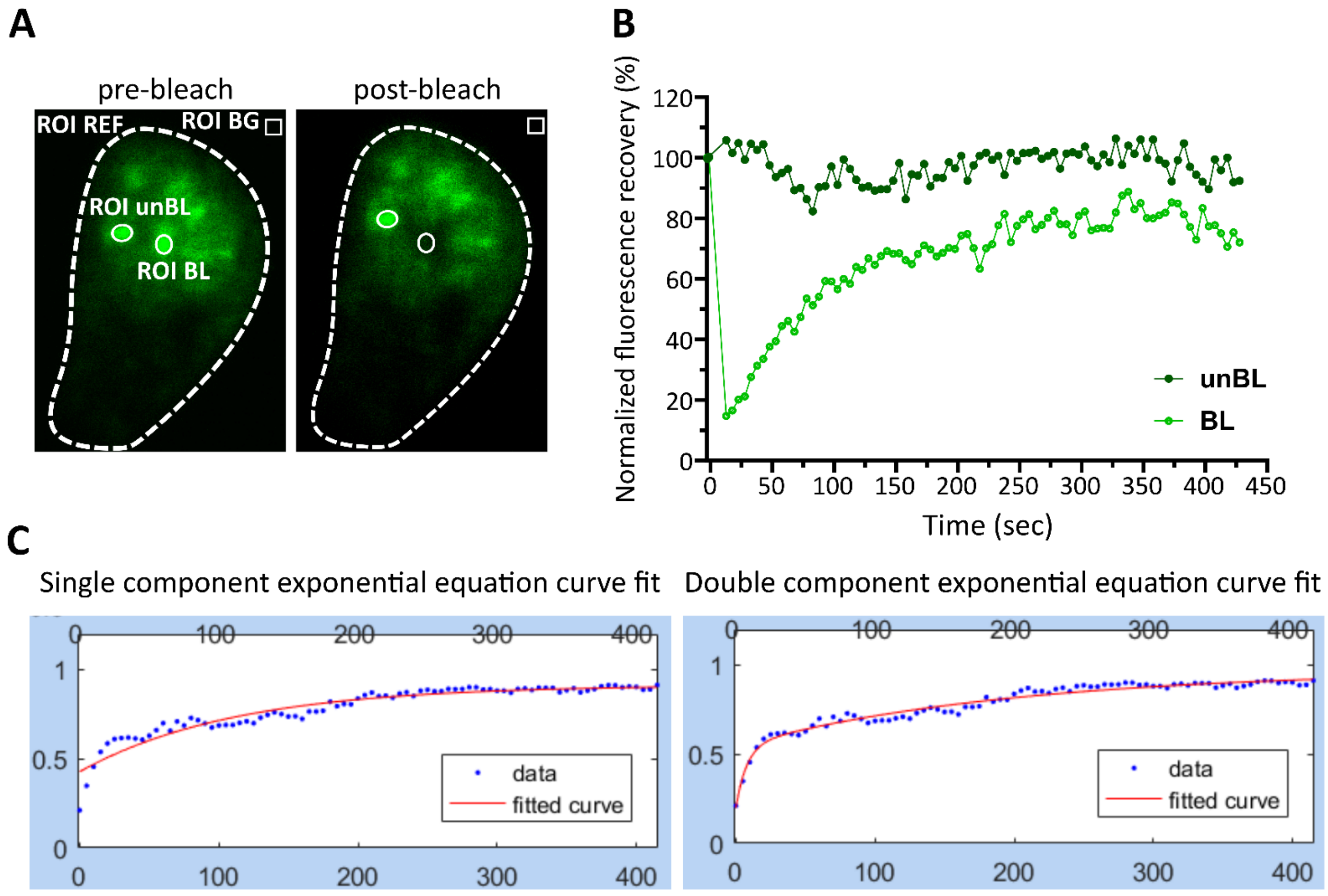

$$I_{fit1} = I_0 - a \cdot e^{-\beta \cdot t}$$

$$I_{fit2} = I_0 - a \cdot e^{-b \cdot t} - \gamma \cdot e^{-\delta \cdot t}$$

Supplementary Figure 3: Fluorescence recovery after photobleaching (FRAP) normalization and curve fitting procedure. (**A**) Representative example of a motor neuron expressing hTDP-43 WT before (pre-bleach, left image) and after photobleaching (post-bleach, right image) of a single nuclear BMC (ROI BL). Unbleached nuclear BMCs (ROI unBL) showed no reduction in fluorescence intensities directly after photobleaching. Background fluorescence intensities (ROI BG) were measured outside the cell, reference area (ROI REF) was the whole cell body. (**B**) Normalized fluorescence recovery values of bleached (BL, light green, bottom curve) and unbleached nuclear BMC (unBL, dark green, top curve) over time. (**C**) Comparing different models to curve fit the normalized fluorescence recovery intensities (blue dots) showed a better curve fit using a double component exponential equation (right image, red line) instead of a single component exponential equation (left image, red line) using EasyFRAP standalone program. The representative example showed the fluorescence recovery curve of a BMC expressing hTDP-43 4FL. Calculated values upon curve fitting: T-half time: 77.6 sec and 26.19 sec, R-square value: 0.91 and 0.97, mobile fraction: 0.85 and 0.97 for single and two component exponential equation respectively.

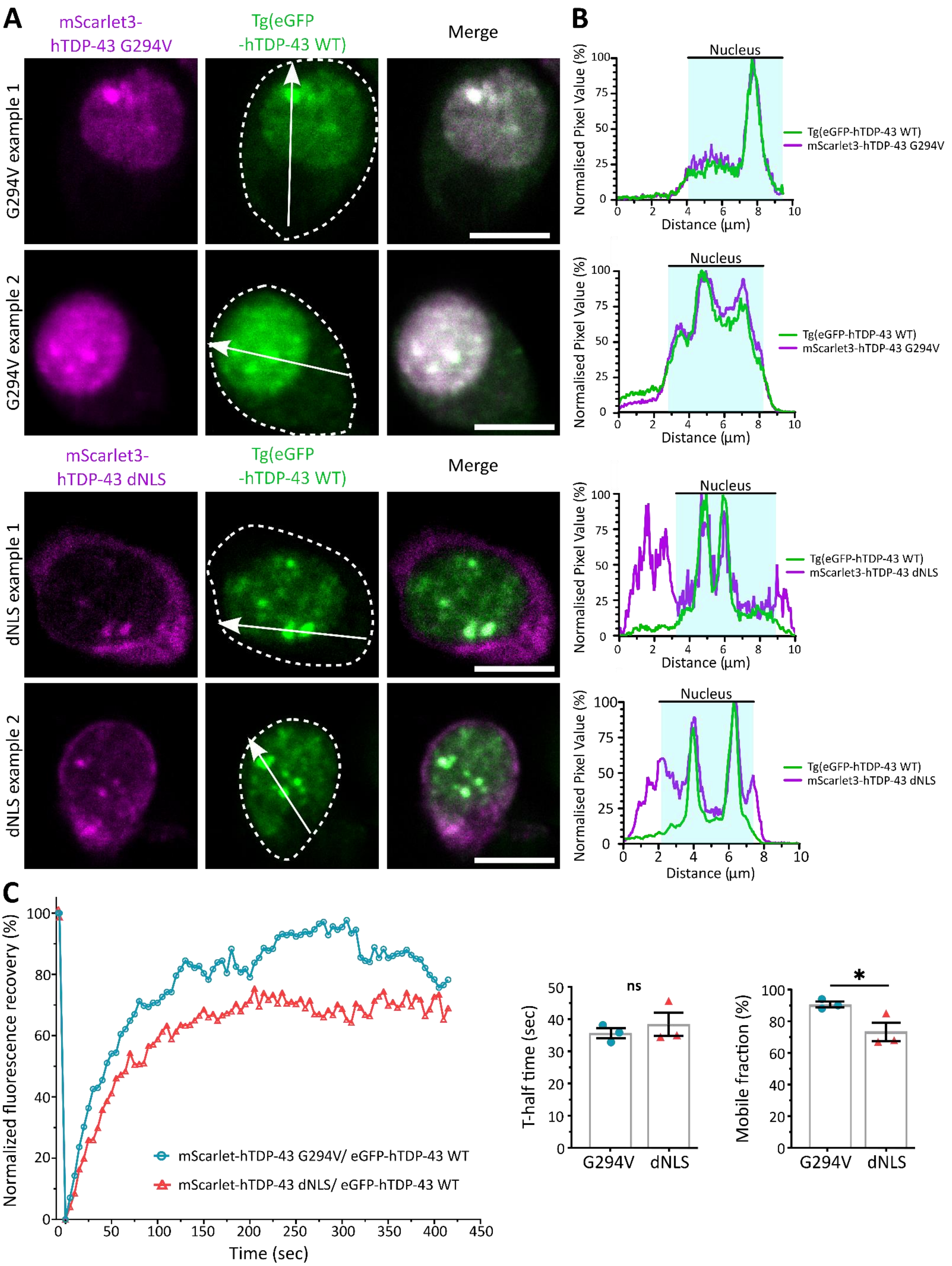
A
mScarlet3-hTDP-43 G294V
Tg(eGFP-hTDP-43 WT)
Merge
G294V example 1
G294V example 2
mScarlet3-hTDP-43 dNLS
Tg(eGFP-hTDP-43 WT)
Merge
dNLS example 1
dNLS example 2
B
Nucleus
Normalised Pixel Value (%)
Distance (µm)
Tg(eGFP-hTDP-43 WT)
mScarlet3-hTDP-43 G294V
mScarlet3-hTDP-43 dNLS
C
Normalized fluorescence recovery (%)
Time (sec)
mScarlet-hTDP-43 G294V/ eGFP-hTDP-43 WT
mScarlet-hTDP-43 dNLS/ eGFP-hTDP-43 WT
T-half time (sec)
ns
G294V
dNLS
Mobile fraction (%)
*

Supplementary Figure 4: hTDP-43 G294V and dNLS form heterotypic BMCs with hTDP-43 WT. (**A**) Representative confocal microscopy images (single confocal plane) of primary motor neurons of the transgenic line *Tg(-3mnx:eGFP-hTDP-43 WT)* co-expressing eGFP-hTDP-43 WT (green) with either mScarlet3-hTDP-43 G294V or dNLS (magenta). Images are contrast adjusted for clarity. White arrows indicate plot profile examples quantified in (B). Dashed line indicates the outer cell soma. (**B**) Fluorescence intensity measurements along the cell axis (white arrow in A) showed heterotypic BMCs consisting of both eGFP-hTDP-43 WT (green) and mScarlet3-hTDP-43 G294V or dNLS (magenta). Pixel values were min/max normalized. (**C**) Mean fluorescence recoveries of BMCs co-expressing eGFP-hTDP-43 WT with mScarlet3-hTDP-43 G294V or dNLS showed no difference in t-half time (G294V: 35.6 sec ± 1.5; dNLS: 38.4 sec ± 3.6) but a decreased mobile fraction (73% ± 6 for mScarlet3-hTDP-43 dNLS; 91% ± 2 for mScarlet3-hTDP-43 G294V); (n= 3 cells from 3 and 2 fish for G294V and dNLS respectively); unpaired t-test (*$p < 0.05$). Data points shown are mean ± SEM. Scale bars represent 5 µm.

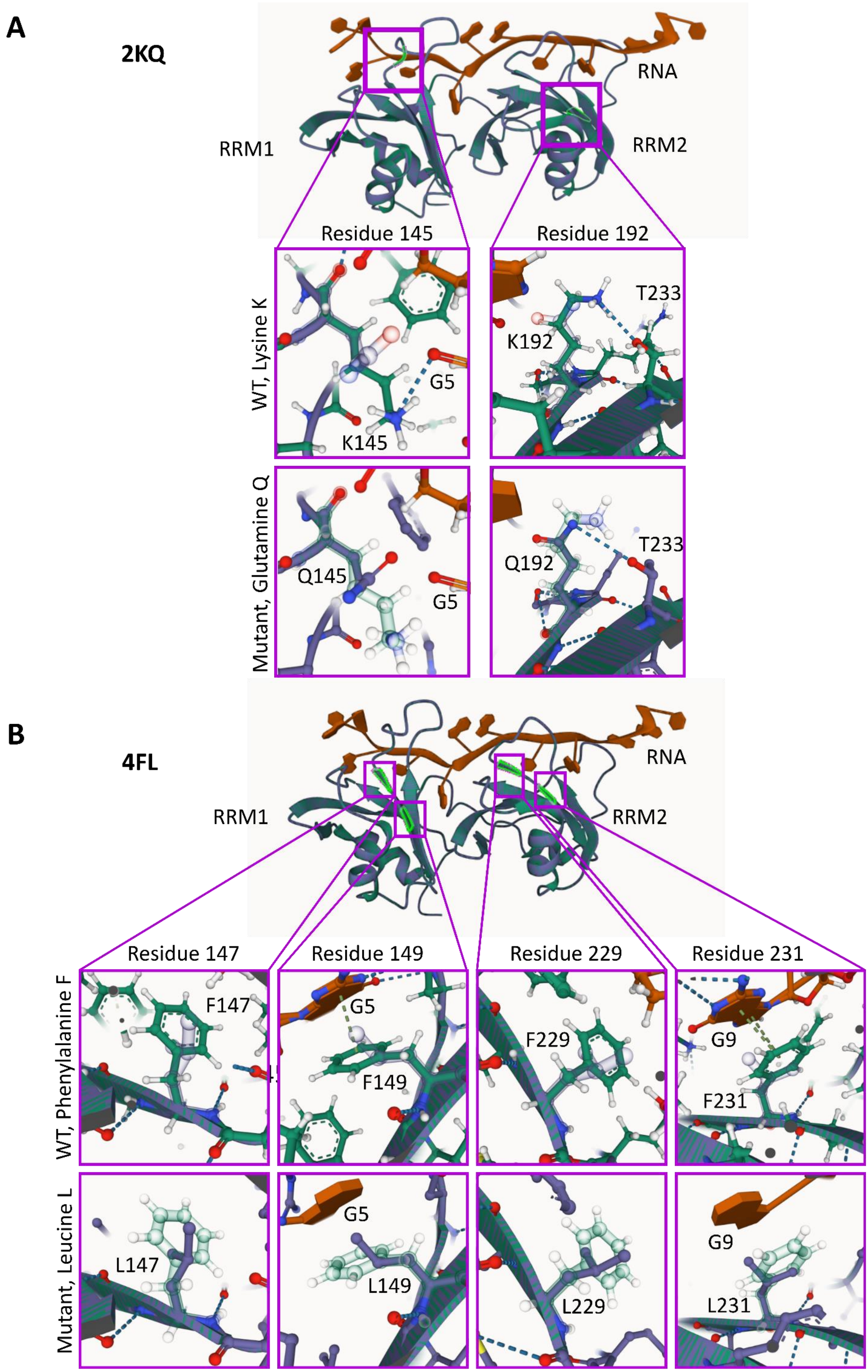
A
2KQ
RNA
RRM1
RRM2
Residue 145
Residue 192
WT, Lysine K
T233
K192
G5
K145
Mutant, Glutamine Q
T233
Q192
Q145
G5
B
4FL
RNA
RRM1
RRM2
Residue 147
Residue 149
Residue 229
Residue 231
WT, Phenylalanine F
F147
G5
F149
F229
G9
F231
Mutant, Leucine L
G5
L147
L149
L229
G9
L231

Supplementary Figure 5: 3D model of hTDP-43 reveals the impact of 2KQ and 4FL mutations on RNA binding. To model the effect of hTDP-43 2KQ and 4FL mutations on its RNA binding ability, we compared changes in bond formation of hTDP-43 $WT_{102\text{-}269}$ fragment binding to UG-rich RNA (in orange) with hTDP-43 $2KQ_{102\text{-}269}$ fragment (A) and hTDP-43 $4FL_{102\text{-}269}$ fragment (B) binding to UG-rich RNA. (**A**) Left panel shows the loss of a hydrogen bond between lysine 145 (K145, green molecule) of hTDP-43 WT and a guanine molecule (G5) of the bound RNA (top right image) upon mutation of the lysine 145 (transparent in background) to glutamine 145 (bottom left image) which can result in altered RNA binding. Right panel shows that the hydrogen bond between lysine 192 (K192, green molecule) and threonine 233 (T233) in hTDP-43 WT (top right image) is unchanged upon lysine 192 (K192, transparent in the background) to glutamine 192 (Q192) mutation (bottom right image). (**B**) Top panel shows phenylalanine residues F147 and F149 in RRM1 and F229 and F231 in RRM2 of hTDP-43 WT. Bottom panel shows the phenylalanine (F) to leucine (L) mutations and the resulting structural changes. F->L mutation at position 149 and 231 revealed a loss of pi-pi stack interaction between the corresponding phenylalanine residues of hTDP-43 and guanine molecule of the bound RNA which can result in altered RNA binding.

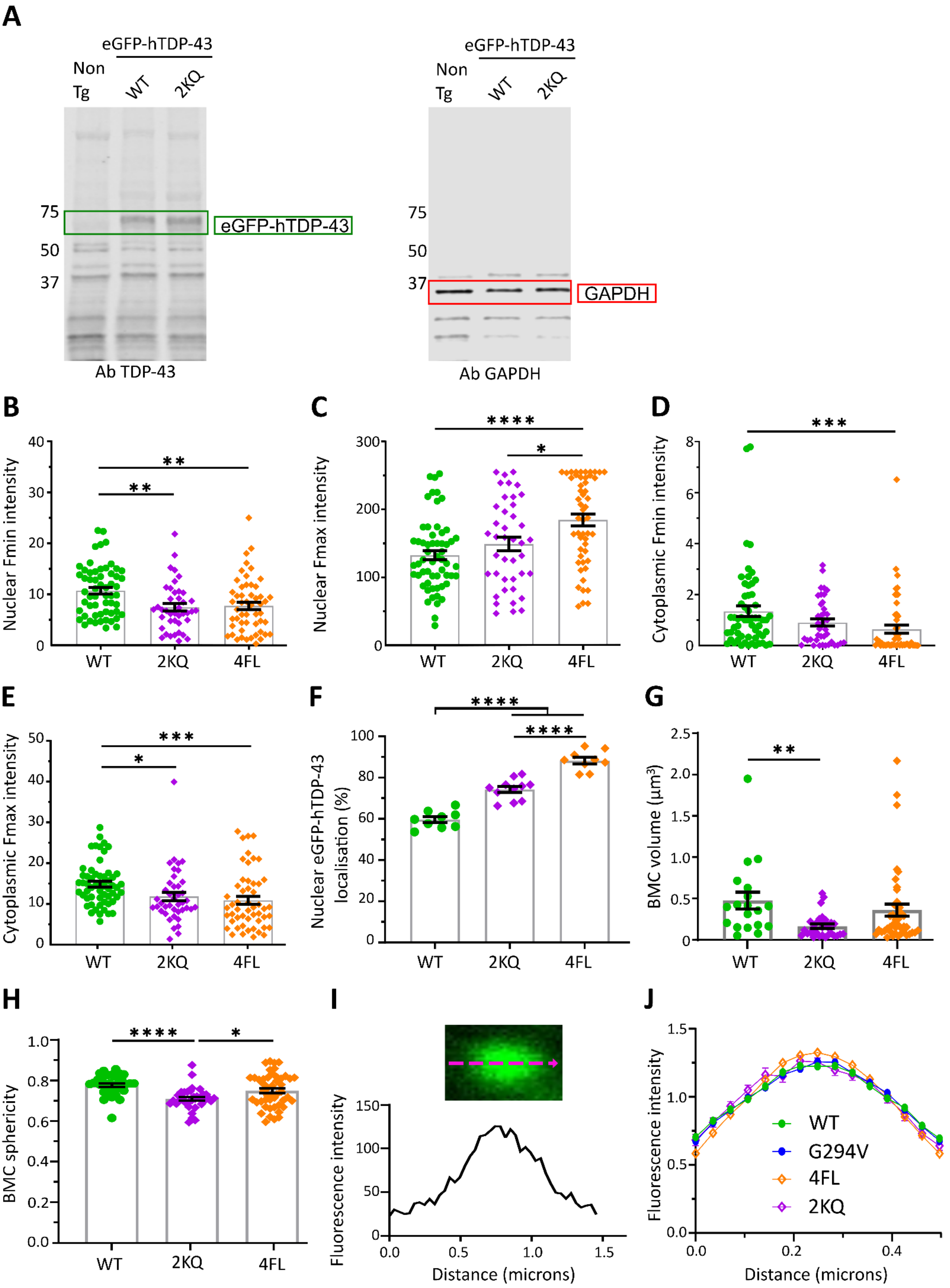
A
eGFP-hTDP-43
Non Tg
WT
2KQ
75
50
37
eGFP-hTDP-43
Ab TDP-43
GAPDH
Ab GAPDH
B
Nuclear Fmin intensity
C
Nuclear Fmax intensity
D
Cytoplasmic Fmin intensity
E
Cytoplasmic Fmax intensity
F
Nuclear eGFP-hTDP-43 localisation (%)
G
BMC volume (µm³)
H
BMC sphericity
I
Fluorescence intensity
Distance (microns)
J
Fluorescence intensity
Distance (microns)
WT
G294V
4FL
2KQ

Supplementary Figure 6: RNA-binding deficient hTDP-43 mutants showed increased nuclear expression and uniform hTDP-43 distribution in biomolecular condensates. (**A**) Western blot analysis of total protein extract of WT TAB embryos and transgenic embryos expressing eGFP-hTDP-43 2KQ using antibodies against TDP-43 and GAPDH (n=18-30 embryos at 3 dpf). (**B-E**) Nuclear and cytoplasmic minimum (Fmin) and maximum (Fmax) fluorescence intensity measurements along a line through the cell's axis (plot profile) for hTDP-43 WT (green), 2KQ (purple) and 4FL (orange), (n= 58, 41 and 51 for WT, 2KQ and 4FL respectively). (**B**) RNA-binding deficient mutant hTDP-43 2KQ (0.9 ± 0.14) and 4FL (0.64 ± 0.16) showed decreased nuclear Fmin intensities compared to WT (1.34 ± 0.21; unpaired One-way ANOVA (**p = 0.0065 and 0.005). (**C**) Only hTDP-43 4FL (184.5 ± 8.8) showed increased nuclear Fmax intensities compared to WT (132.5 ± 6.9) and 2KQ (149 ± 10.1); unpaired One-way ANOVA (*p = 0.0269, ****p ≤ 0.0001). (**D**) hTDP-43 4FL (0.64 ± 0.16) showed significantly decreased cytoplasmic Fmin intensities compared to WT (1.3 ± 0.2) and 2KQ (0.9 ± 0.14); unpaired One-way ANOVA (***p = 0.0002, *p = 0.0143). (**E**) Cytoplasmic Fmax intensities were increased in hTDP-43 WT (14.84 ± 0.7) compared to 2KQ (11.84 ± 1) and 4FL (10.89 ± 0.99) mutants; unpaired One-way ANOVA (*p = 0.0143, ***p = 0.0002). (**F**) 3D volume quantification of eGFP-hTDP-43 and H2BmCerulean3 expressing motor neurons showed a higher nuclear localization for hTDP-43 2KQ (74% ± 1.5) and 4FL (88.3% ± 1.6) compared to WT (59.6% ± 1.4) (n= 9, 11 and 9 motor neurons for WT, 2KQ, and 4FL respectively); unpaired one-way ANOVA (****p ≤ 0.0001). (**G**) Quantification of 3D rendered BMCs (Imaris) showed a significant decrease in volume for the hTDP-43 2KQ mutant (0.16 μm^3 ± 0.03) in comparison to hTDP-43 WT (0.47 μm^3 ± 0.10) and 4FL mutant (0.35 μm^3 ± 0.07), (n=19, 30, 42 BMCs of 7, 8, 8 motor neurons for WT, 2KQ and 4FL respectively); unpaired One-way ANOVA (*p = 0.0162). (**H**) Nuclear 3D rendered BMCs (Imaris) expressing hTDP-43 WT (0.78 ± 0.009), 2KQ (0.71 ± 0.009) and 4FL (0.75 ± 0.119) were comparably spherical in shape (n=32, 34 and 47 BMCs for WT, 2KQ and 4FL respectively); unpaired One-way ANOVA (*p = 0.0292, ****p ≤ 0.0001). (**I**) Representative example image of fluorescence intensity values of a plot profile along a line through a single nuclear BMC expressing hTDP-43 WT showed a uniform eGFP-hTDP-43 expression with a maximum in fluorescence in the center. (**J**) Mean fluorescence intensities of plot profiles of single nuclear BMCs showed uniform eGFP-hTDP-43 expression for hTDP-43 wildtype and all mutant versions (n= 20, 17, 10 and 20 BMCs for WT, G294V, 2KQ and 4FL respectively). Data points shown are mean ± SEM.

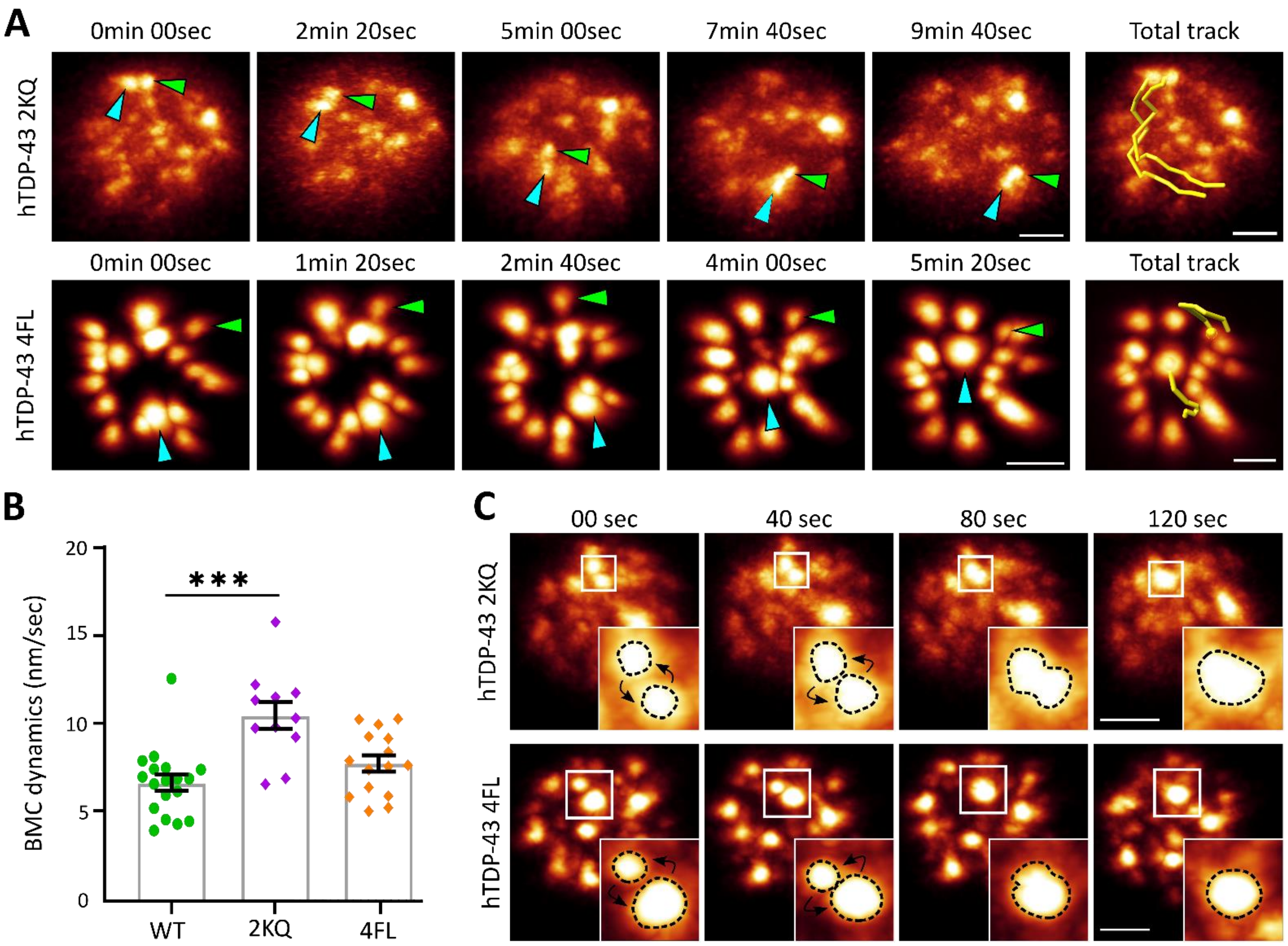

Supplementary Figure 7: Nuclear RNA-binding deficient BMCs are dynamic and undergo spontaneous fusion and fission. (**A-B**) High-resolution time-lapse imaging revealed that BMCs are dynamic within the nucleus. Nuclear BMCs expressing hTDP-43 2KQ (10.6 nm/sec ± 0.8) showed a significant increase in speed when compared to hTDP-43 WT (6.8 nm/sec 0.5), (n= 18, 11 and 15 BMCs for WT, 2KQ and 4FL respectively); unpaired One-way ANOVA (***p = 0.6* 10^{-3}). Data points shown are mean ± SEM. Time-lapse videos attached in supplementary source. (**C**) Examples of hTDP-43 2KQ and 4FL positive BMCs undergoing spontaneous fusion and fission over a short time. Time-lapse videos attached in supplementary source. Scale bar represents 2 µm.

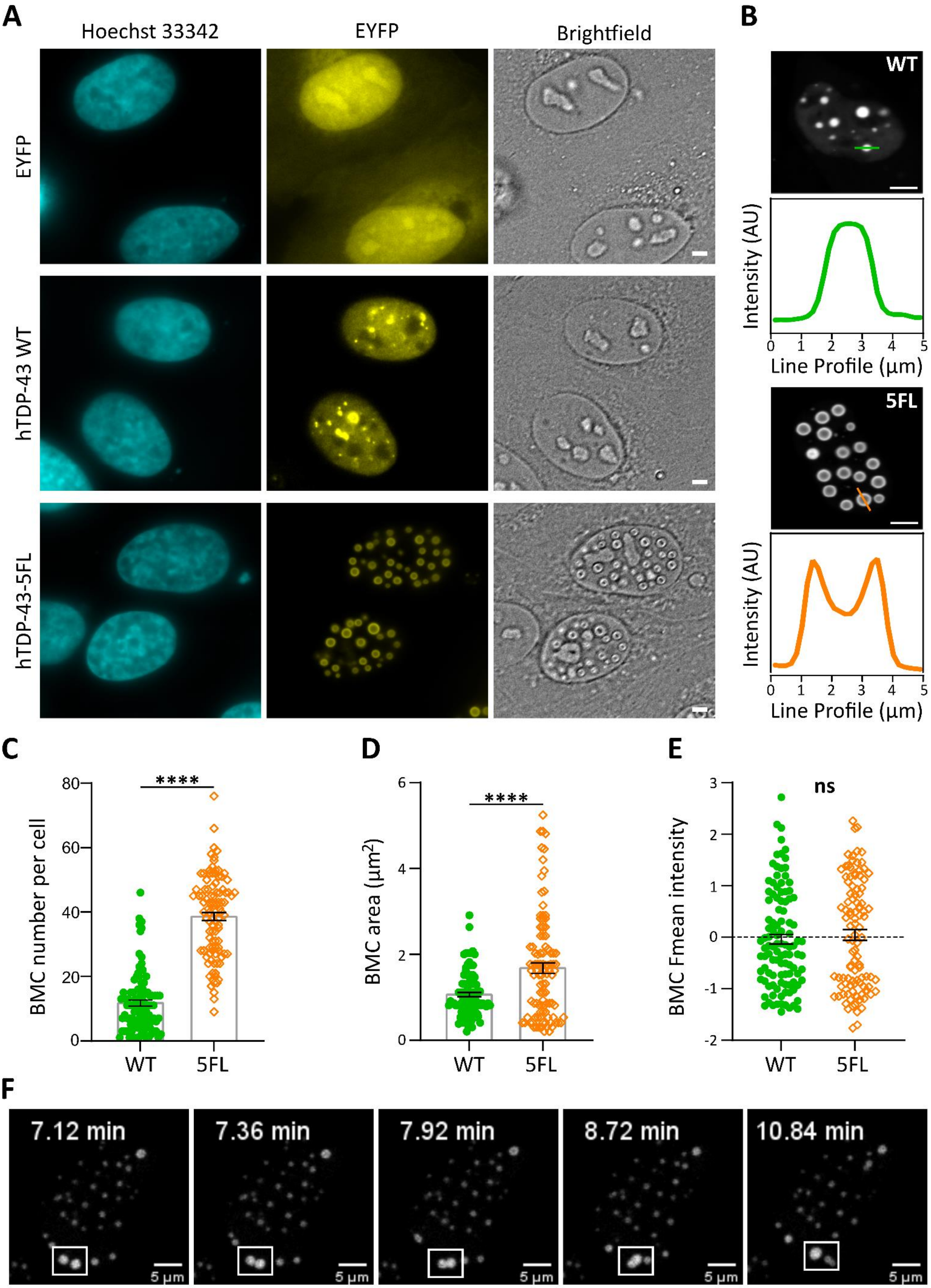
A
Hoechst 33342
EYFP
Brightfield
EYFP
hTDP-43 WT
hTDP-43-5FL
B
WT
Intensity (AU)
Line Profile (µm)
5FL
Intensity (AU)
Line Profile (µm)
C
BMC number per cell
****
WT
5FL
D
BMC area (µm2)
****
WT
5FL
E
BMC Fmean intensity
ns
WT
5FL
F
7.12 min
7.36 min
7.92 min
8.72 min
10.84 min
5 µm

Supplementary Figure 8: Mutant hTDP-43 5FL forms anisotropic BMCs in human cells. (A) Representative images of U2OS cells expressing EYFP, TDP-43-WT, or TDP-43-5FL (Hoechst 33342 in cyan, EYFP in yellow, brightfield in greyscale). **(B)** Fluorescence intensity line profiles of individual BMCs expressing hTDP-43 WT and 5FL. (**C**) The number of nuclear BMCs expressing hTDP-43 5FL (38.6 ± 1.3) is significantly higher when compared to WT (11.7 ± 0.9), (n=102 and 100 cells for 5FL and WT respectively); Welch's t-test (****p ≤ 0.0001). (**D**) Increased area of nuclear BMCs expressing hTDP-43 5FL (1.7 μm^2 ± 0.1) compared to WT (1.1 μm^2 ± 0.05), (n=101 and 103 BMCs for 5FL and WT respectively); Welch's t-test (****p ≤ 0.0001). (**E**) Normalized mean fluorescence intensities of BMCs expressing hTDP-43 WT (0.04 SD below the mean ± 0.09) and 5FL (0.04 SD above the mean ± 0.1) are similar (n= 101 and 108 BMCs for WT and 5FL respectively); Welch's t-test (ns=non-significant). **(F)** Time-lapse microscopy of a representative single cell expressing hTDP-43 5L showing the fusion of two BMCs (white box). Data points shown are mean ± SEM. All scale bars shown are 5 µm.

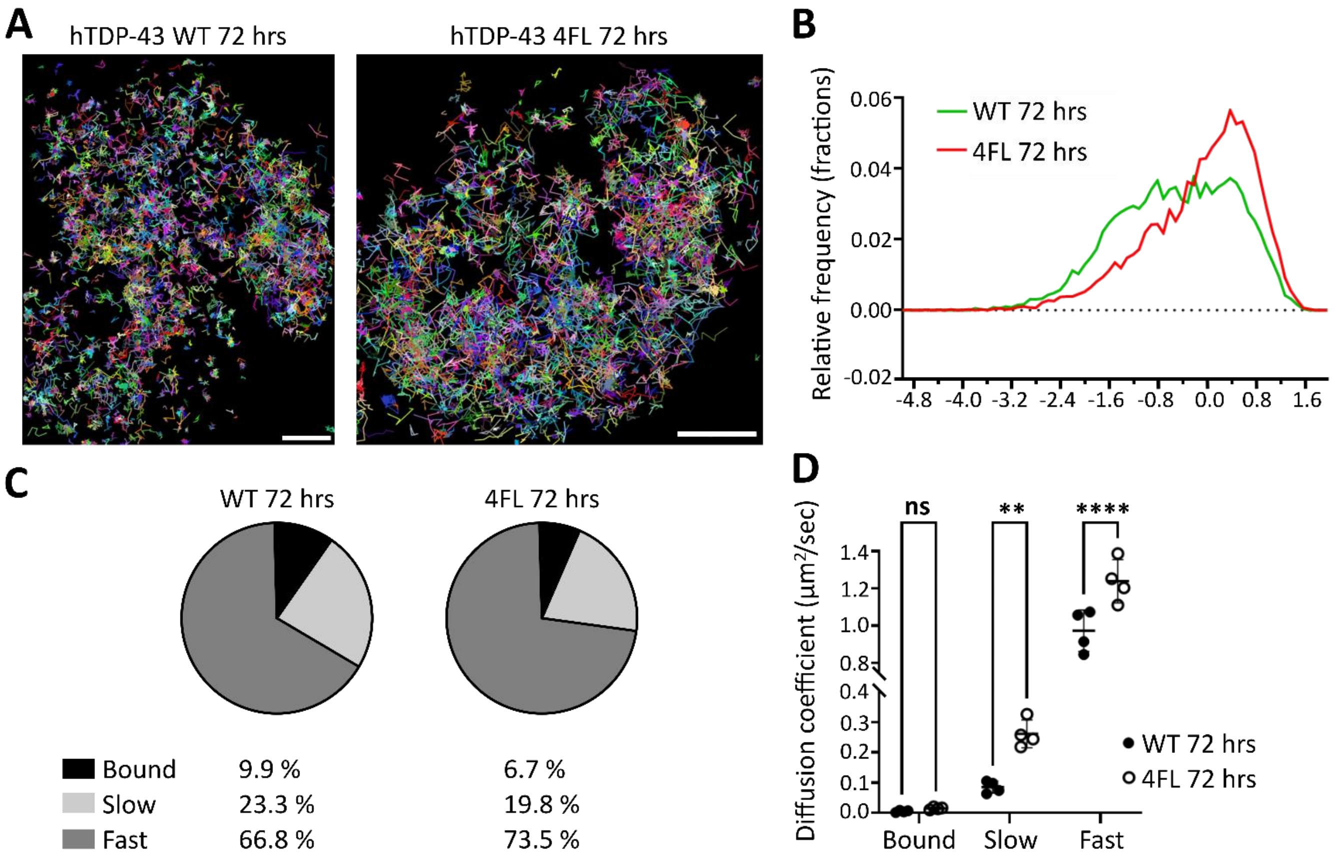

Supplementary Figure 9: Single Molecular Tracking of hTDP-43 WT and 4FL molecules showed an altered motility and diffusion profile for 4FL variant in HeLa cells. (**A**) Representation of Halo-tagged hTDP-43 trajectories in the nucleus of HeLa cells using HILO microscopy. Scale bar 2 µm. (**B**) Quantification of the mobility profile of HALO-hTDP-43 WT (in green) and 4FL (in red) using diffusion coefficient for all cells ($\mu m^2.s^{-1}$). Mean values ± SD are shown for n= 4 cells each. (**C**) Diagrams representative of the bound, slow, and fast fraction for each variant showed an increase of the fast diffusion for 4FL (73.50% vs 66.8%) and a decrease in the bound (6.7% vs 9.9%) and slow (19.8% vs 23.3%) fraction when compared to TDP-43 WT. (**D**) Representation of bound, slow, and fast diffusion coefficient (in μm^2/sec) using a three-state model. TDP-43 4FL showed an overall higher slow (0.26 ± 0.05) and fast diffusion coefficient (1.2 ± 0.1) compared to WT (0.09 ± 0.02 slow and 0.97 ± 0.1 fast), (n= 4 cells each); 2-way ANOVA (**$p < 0.01$, ****$p \leq 0.0001$).

Supplementary Tables

Supplementary Table S1: Statistical analysis of heterotypic BMCs

	mScarlet3-hTDP-43 G294V: eGFP-hTDP-43 WT	mScarlet3-hTDP-43 dNLS: eGFP-hTDP-43 WT
Number of values	10	10
	**Pearson's coefficient within ROI volume** (Linear correlation between two channels' pixels)	
Mean	0.76	0.33
Standard Deviation	0.15	0.22
Standard Error of the Mean	0.05	0.07
95% Confidence Interval	0.65 to 0.87	0.18 to 0.49
	**One Sample T-Test:** Null hypothesis: no correlation, mean is 0 Alternative hypothesis: true mean is greater than 0	
Test Statistic[1]	16.3	4.9
Degrees of Freedom[2]	9	9
P value[3]	2.76E-08	4.30E-04

1. Difference between mean and null hypothesis divided by standard error
2. N-1, number of values in the final calculation that are free to vary
3. If null hypothesis true, probability of observing a test statistic at least as extreme

Supplementary Table S2: Multistate kinetic model statistics (related to Figure 6)

**Two-state kinetic model**						
**Protein**	**Dbound μm²/s**	**Dfree μm²/s**	**Fbound (%)**	**Ffree (%)**		
WT	0.03	0.907	36.2	63.8		
4FL	0.046	1.017	17.9	82.1		
2KQ	0.055	1.001	17.5	82.5		
**Three-state kinetic model**						
**Protein**	**Dbound μm²/s**	**Dslow μm²/s**	**Dfast μm²/s**	**Fbound (%)**	**Fslow (%)**	**Ffast (%)**
WT	0.018	0.279	1.315	27.4	27.2	45.5
4FL	0.021	0.339	1.296	11.6	24.9	63.4
2KQ	0.024	0.402	1.388	10.7	31.8	57.4

Supplementary Table S3: Summary of phase separation quantification *in vivo* and *in vitro*

		RNA-binding deficient mutants			ALS variant
	**WT**	**2KQ**	**4FL**	**5FL**	**G294V**
Nuclear localisation (%)	60	**74**	**88**	-	61
BMC number	21	22	26	-	19
BMC area (μm^2)	0.159	*0.115*	*0.145*	-	0.152
BMC volume (μm^3)	0.47	*0.16*	0.35	-	0.42
BMC dynamic (nm/sec)	6.8	**10.6**	8	-	6.7
Fmean intensity ratio (diffuse/tot nucleus)	0.96	0.86	*0.66*	-	0.98
T-half time (sec) *in vivo*	50.9	*18.4*	*29.8*	-	51.8
T-half time (sec) *in vitro*	44.3	-	-	*20.3*	-
Mobile fraction (%) *in vivo*	83	82	81	-	81
Mobile fraction (%) *in vitro*	33	-	-	**51**	-
Fraction of mobile molecules *in vitro*	54	77	79	-	-
Long dwell time (sec)	8	**11**	**12**	-	-

Values in bold indicate significant higher values.

Values in italic indicate significant lower values.
