# Supplementary material for: RNA-binding properties orchestrate TDP-43 homeostasis through condensate formation in vivo
Source: Nucleic Acids Res. 2024 Feb 21;52(9):5301–19. doi: 10.1093/nar/gkae112 (PMC11109982; doi:10.1093/nar/gkae112)
Supplement: gkae112_Supplemental_files [file gkae112_supplemental_files.zip › Supplementary Video Legends.docx]

**Supplementary Videos:**

**Supplementary Video 1: Time-lapse imaging of nuclear TDP-43-WT BMCs inside motor neurons *in vivo***

Time-lapse video of mobile BMCs in single motor neurons expressing hTDP-43 G294V. Images represent z-stacks of whole motor neurons taken every 40 seconds.

**Supplementary Video 2: Time-lapse imaging of nuclear TDP-43-G294V BMCs inside motor neurons *in vivo***

Time-lapse video of mobile BMCs in single motor neurons expressing hTDP-43 G294V. Images represent z-stacks of whole motor neurons taken every 40 seconds.

**Supplementary Video 3: Time-lapse imaging of fusion and fission events of nuclear TDP-43-WT BMCs *in vivo***

Time-lapse video of BMCs in single motor neurons undergoing fusion and fission for hTDP-43 WT. Images represent z-stacks of whole motor neurons every 40 seconds.

**Supplementary Video 4: Time-lapse imaging of fusion and fission events of nuclear TDP-43-G294V BMCs *in vivo***

Time-lapse video of BMCs in single motor neurons undergoing fusion and fission for hTDP-43 G294V. Images represent z-stacks of whole motor neurons every 40 seconds.

**Supplementary Video 5: Time-lapse imaging of nuclear TDP-43-2KQ BMCs inside motor neurons *in vivo***

Time-lapse video of mobile BMCs in single motor neurons expressing acetylation mimicking hTDP-43 2KQ. Images represent z-stacks of whole motor neurons taken every 40 seconds.

**Supplementary Video 6: Time-lapse imaging of nuclear TDP-43-4FL BMCs inside motor neurons *in vivo***

Time-lapse video of mobile BMCs in single motor neurons expressing RNA-binding deficient hTDP-43 4FL. Images represent z-stacks of whole motor neurons taken every 40 seconds.

**Supplementary Video 7: Time-lapse imaging of fusion and fission events of nuclear TDP-43-2KQ BMCs *in vivo***

Time-lapse video of BMCs in single motor neurons undergoing fusion and fission for acetylation mimicking hTDP-43 2KQ. Images represent z-stacks of whole motor neurons every 40 seconds.

**Supplementary Video 8: Time-lapse imaging of fusion and fission events of nuclear TDP-43-4FL BMCs *in vivo***

Time-lapse video of BMCs in single motor neurons undergoing fusion and fission for RNA-binding deficient hTDP-43 4FL. Images represent z-stacks of whole motor neurons every 40 seconds.
